# From Surface to Subsurface: Diversity, Composition, and Abundance of Sessile and Endolithic Bacterial, Archaeal, and Eukaryotic Communities in Sand, Clay and Rock Substrates in the Laurentians (Quebec, Canada)

**DOI:** 10.3390/microorganisms10010129

**Published:** 2022-01-08

**Authors:** Julia Meyer, Sheri Zakhary, Marie Larocque, Cassandre S. Lazar

**Affiliations:** 1 Department of Biological Sciences, University of Quebec at Montreal, C.P. 8888, Succ. Centre-Ville, Montréal, QC H3C 3P8, Canada; meyer.julia@courrier.uqam.ca (J.M.); sherizakhary@gmail.com (S.Z.); 2 Department of Earth and Atmospheric Sciences and GEOTOP, University of Quebec at Montreal, C.P. 8888, Succ. Centre-Ville, Montréal, QC H3C 3P8, Canada; larocque.marie@uqam.ca

**Keywords:** terrestrial subsurface, genomics, bacteria, archaea, eucaryote, planktonic, microbial interactions

## Abstract

Microbial communities play an important role in shallow terrestrial subsurface ecosystems. Most studies of this habitat have focused on planktonic communities that are found in the groundwater of aquifer systems and only target specific microbial groups. Therefore, a systematic understanding of the processes that govern the assembly of endolithic and sessile communities is still missing. This study aims to understand the effect of depth and biotic factors on these communities, to better unravel their origins and to compare their composition with the communities detected in groundwater. To do so, we collected samples from two profiles (~0–50 m) in aquifer sites in the Laurentians (Quebec, Canada), performed DNA extractions and Illumina sequencing. The results suggest that changes in geological material characteristics with depth represent a strong ecological and phylogenetical filter for most archaeal and bacterial communities. Additionally, the vertical movement of water from the surface plays a major role in shallow subsurface microbial assembly processes. Furthermore, biotic interactions between bacteria and eukaryotes were mostly positive which may indicate cooperative or mutualistic potential associations, such as cross-feeding and/or syntrophic relationships in the terrestrial subsurface. Our results also point toward the importance of sampling both the geological formation and groundwater when it comes to studying its overall microbiology.

## 1. Introduction

In the shallow terrestrial subsurface (<50 m below surface), microorganisms play an important role in soil nutrition cycling, soil respiration, soil formation, ecosystem biochemical processes, contaminant degradation, as well as groundwater maintenance [1,2]. In recent years, many subsurface inventories have surveyed the distribution patterns or processes that govern microbial community assembly [1,2]. However, due to the difficulty in accessing rocks and sediments below ground, most of these inventories have focused solely on the planktonic communities that live suspended in the aquifer groundwater of this habitat [3]. Yet, endolithic and sessile communities that live in rocks and are attached to sediments constitute the majority of cells in the subsurface [4]. Consequently, most of these subsurface inventories bear the risk of misconceptions [3].

Depth plays a major role in shaping microbial communities in subterranean ecosystems [5,6,7,8,9,10,11,12]. Depth is also related to the mechanisms of microbial community formation because vertical movements of water from the surface bring microorganisms into the terrestrial subsurface [13]. However, despite the existing literature on the effect of depth on microbial communities, our overall understanding remains incomplete. Primarily, studies on this subject are still limited and most of them describe the effect of depth on large spatial scales (by comparing the surface with the subsurface), or by focusing exclusively on near-surface soil horizons [5,6,14]. Studies also often target specific microbial domains (e.g., archaea, bacteria, or eukaryote), making it difficult to gain a systematic understanding of the total microbial community diversity and the interactions between organisms composing them [2,14]. Moreover, although different microbial structural characteristics have been examined in a previous study, few studies have simultaneously studied the effect of depth on absolute abundance, phylogenetic diversity, taxonomic diversity, and composition [14]. Yet, these characteristics provide complementary information. Analysing all these characteristics, allows us to get more insight into the processes at the origin of the assembly of communities by making it possible to quantify the importance of different ecological and evolutionary processes such as dispersion, competition, and filtration of the environment. In addition, learning the origins of microbial communities may not only significantly improve our current understanding of how shallow subsurface microbial communities are formed, but could also inform on the consequences of some human activities that affect the soil (e.g., agricultural inputs) [15].

Therefore, the objectives of this study are to (1) determine which factors govern microbial community diversity in samples from surface to the bedrock (i.e., shallow subsurface), and (2) estimate the proportion of subsurface communities that are formed through vertical fluid fluxes originating from the surface, in two Laurentian sites harboring aquifers. We analyzed abiotic factors such as the physicochemical, geochemical, and mineralogical characteristics of the geological material with depth, but also biotic factors such as potential interactions between organisms. The total microbial community was studied though sequencing of archaeal and bacterial 16S rRNA genes, as well as 18S rRNA eukaryotic genes.

## 2. Materials and Methods

### 2.1. Study Sites 

**Figure 1:**
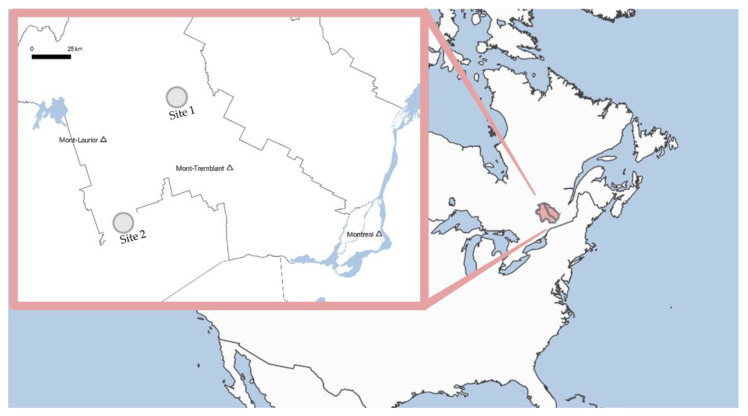
Location of the two study sites. Both sites are to the northwest of Montreal in the Laurentians region (Quebec, Canada).

Two sites in the Laurentians region (Quebec, Canada) were selected for this study. The first site is in the municipality of Ascension (46°36′42.66″ North, 74°47′3.911″ East) and the second site is in the municipality of Notre-Dame-du-Laus (46°0′59.148″ North, 75°34′37.667″ East) (Figure 1). Both sites differ in terms of the size and relative abundance of mineral particles. At site 1 (Ascension), the geological material was mostly composed of sand and had a smaller amount of clay compared to site 2 (Notre-Dame-du-Laus) (Supplementary Tables S1 and S2). Both sites are located in a forested area. At site 1, the vegetation type is dominated by resinous trees mainly composed of black spruce. Site 2 is also located in a resinous tree dominated forest, but pine is the dominant species. Well-drained loam is the soil type in both sites. For site 1, climatic data from the period of 1981–2010 were taken from the station located 25 km southward [16]. Mean annual precipitation is 1028.9 mm and annual average temperature is 4 °C. At site 2, climate data from the closest weather station for which a 1981–2010 climatic normal was available is located 33 km west [17]. Mean annual precipitation is 939.9 mm and annual average temperature is 4.7 °C. Samples were acquired during a regional scale aquifer characterization project (Department of Earth and Atmospheric Sciences, UQAM). During this project, two new wells were drilled using a double rotation drill in a destructive mode during the summer of 2019. Bedrock was reached at 20.73 m at site 1 (drilling stopped at 42.06 m), and 48.77 m at site 2 (drilling stopped at 49.38 m). 

### 2.2. Sampling

**Figure 2:**
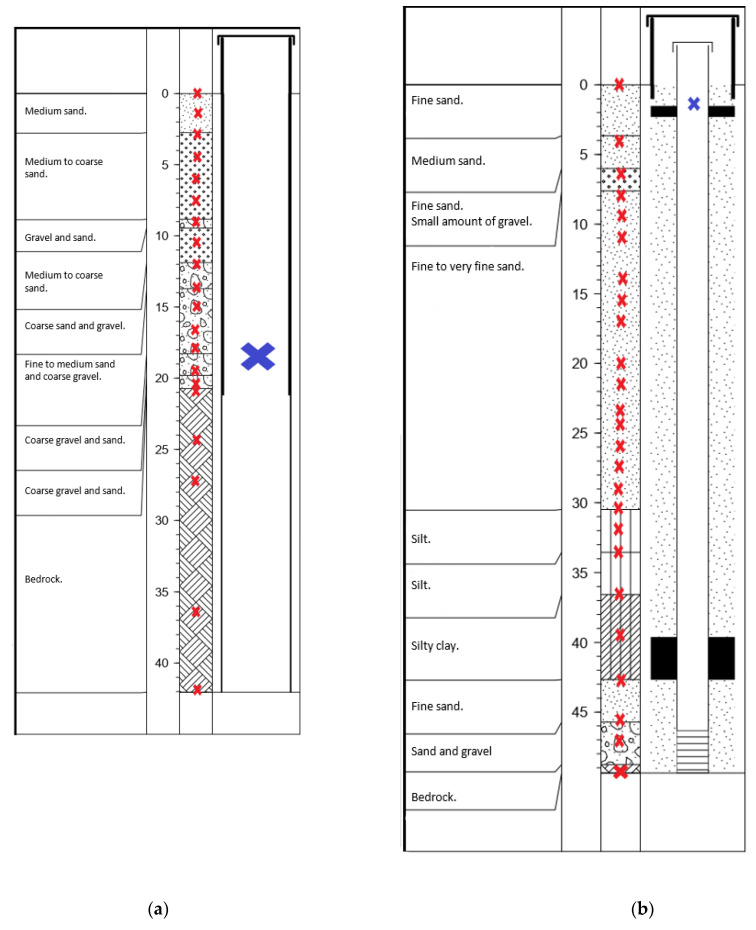
Diagrams of the 15.2 cm diameter boreholes, showing the depth at which samples of rock-associated (red crosses, sediments samples during drilling) and planktonic (blue crosses, samples in the well water after drilling) subsurface communities were taken (**a**) at site 1 (Ascension) and (**b**) site 2 (Notre-Dame-du-Laus).

A total of 46 samples were collected, i.e., 20 samples at site 1 (Figure 2a) and 26 samples at site 2 (Figure 2b). Sampling was carried out vertically from the surface down to the bedrock, from the material extracted during the drilling. Estimation of depth for each sample was based on the operator’s information during drilling. It is estimated that these depths are precise to ±0.5 to 1 m. The soil, sediment, and crushed bedrock material rising from the subsurface were collected in sterile 50 mL Falcon tubes on the same day as the drilling. To minimize potential contamination, we sub-sampled them by taking the materials in the center of the container used for sampling. We collected water used for drilling (from the surface water of a local stream) in a 50 mL Falcon tube, to identify potential contamination from microbial communities potentially introduced into the samples during drilling. Groundwater samples were collected in the newly drilled wells at site 1 (18 m depth) and site 2 (1.75 m depth), using a submersible pump (12 V/24 V Mini-Monsoon, Waterra, Canada). It is important to underline that the sampled groundwater from site 1 represents a mix of water coming from the open borehole, i.e., between 20.73 and 42.06 m. 

### 2.3. Chemical Characteristics of Geological Material and Water Samples

Geological material texture was determined in the field by touch [18]. Prior to further analysis, subsamples of geological materials were dried at 60 °C for 48 h and ground into a homogeneous powder using a mortar and pestle. Soil pH was measured in triplicate in a 1:2 (*w/v*) soil/water ratio using an Accumet XL600 pH meter (Fisher Scientific, Waltham, MA, USA) after 1 h of incubation [19]. Then, analyses for total nitrogen content (TN), total carbon content (TC), total organic carbon content (TOC) and total inorganic carbon (TIC) of geological material were conducted at the light stable isotope geochemistry laboratory of the GEOTOP center at UQAM using the NC 2500 elemental analyzer (Carlo Erba, Milan, Italy). TOC content was determined after pretreatment of the samples with 5% HCl to remove inorganic carbon. The mineral composition of the samples was determined at the research center NanoQAM (University of Quebec in Montreal, Quebec, Canada) with the X-ray diffraction (XRD) method using the X-ray diffractometer D8 Advance (Bruker, Billlerica, MA, USA). The instrument was adapted with a copper tube (Cu K-α = 154,178 Å) and samples were scanned from 5° to 70° 2-θ with a step of 0.02° 2-θ. The resulting diffractograms were converted to percentage by weight of minerals using the powdR program (Retrieved 10 December 2020, from https://CRAN.R-project.org/package=powdR, accessed on 23 December 2021) [20] and quartz as internal standard. For the water samples, only the pH was determined. To do so, we used an Oakton pH meter (Waterproof pH/DO 450 Meter, Oakton, CA, USA).

### 2.4. DNA Extraction

DNA was extracted from 10 g of samples following the instructions of the commercial DNeasy PowerMax Soil Kit (Qiagen, Hilden, Germany) with some modifications [21]. Obtaining representative DNA extracts proved very challenging due to the low cellular biomass, the adsorption of cells to geological material, and the frequent co-extraction of enzymatic inhibitors [3,22]. Therefore, following Direito et al. [23] we optimized the kit by using a 1 M of NaH_2_PO_4_/Na_2_HPO_4_ buffer in the initial steps of the kit manufacturer’s protocol [3]. One control sample was tested for detecting extraction-process contamination by applying sterilized water to the DNA extraction kit. The water samples (drilling fluid and groundwater from sites 1 and 2) were filtered through a sterile 0.2 µm polyether sulfone filter (Sartorius, Midisart, Germany). DNA was extracted using the DNeasy PowerWater Kit (Qiagen, Hilden, Germany). The obtained DNA extracts were stored at −20 °C.

### 2.5. PCR Amplification and Illumina Sequencing

Sequencing was performed at the Center of Excellence in Research on Orphan Disease–Fondation Courtois (CERMO-CF) at UQAM. DNA extracted from each sample was amplified with universal primers that target the hypervariable V3-V4 region of the bacterial 16S rRNA genes, the V3-V4-V5 region of the archaeal 16S rRNA genes, and the V7 region of the eukaryotic 18S rRNA genes. For this, the primer pair B341F (5′-CCT ACG GGA GGC AGC AG -3′) [24]—B785R (5′- GAC TAC CGG GGT ATC TAA TCC -3′) [25], A340F (5′-CCC TAC GGG CYC CAS CAG-3′) [26] - A915R (5′-GTG CTC CCC CGC CAA TTC CT-3′) [27] and E960F (5′-GGCTTAATTTGACTCAACRCG-3′) [28]—NSR1438R (5′-GGGCATCACAGACCTGTTAT-3′) [29] were used. PCR amplification was carried out using the UCP HiFidelity PCR kit (Qiagen, Hilden, Germany). Amplification was performed under the following PCR conditions: initial denaturation at 98 °C for 30 s, denaturation at 98 °C for 30 s, primer annealing for 30 seconds (at 57 °C for bacteria, 58 °C for archaea and 56.6 °C for eukaryote), extension at 72 °C for 1 min, and a final extension at 72 °C for 10 minutes. The denaturation, annealing and extension steps were repeated 33 times for bacteria and 40 times for both archaea and eukaryote. Sequencing was performed using an Illumina MiSeq (2 × 300) and the MiSeq Reagent Kit v.3 (600 cycles, Illumina), according to manufacturer’s instructions. The obtained sequences were deposited in the National Center for Biotechnology Information (NCBI) under the BioProject ID: PRJNA758373.

### 2.6. Digital PCR

Digital PCR amplification used for the estimation of absolute microbial abundance was challenging because of the overall low microbial biomass, especially for archaeal and eukaryotic genes. Therefore, we only show digital PCR results for the 16S rRNA genes of the bacteria (the number for archaea and eukaryotes were below the quality threshold). These analyses were performed using the QuantStudio 3D Digital PCR (ThermoFisher, Waltham, MA, USA) instrument and the QuantStudio 3D PCR Master Mix v2 (ThermoFisher, USA). The primers used were B341F (5′-CCT ACG GGA GGC AGC AG-3′) [24]—B785R (5′-GAC TAC CGG GGT ATC TAA TCC-3′) [25] and the reaction procedure was as follows: 96 °C for 10 min; 39 PCR cycles of 56 °C for 3 min, annealing at 56 °C for 3 min, then 98 °C for 30 s, and a final extension at 56 °C for 2 min. The dPCR results were expressed as gene copies per gram of geological material (copies g^−1^).

### 2.7. Sequence Processing

Sequence data was processed using the DADA2 Pipeline (v.1.18.1) (Retrieved 29 June 2021, from https://benjjneb.github.io/dada2/tutorial_1_8.html, accessed on 23 December 2021) described by Callahan et al. [30]. Based on the quality profiles generated for both forward and reverse reads, we chose to truncate the bacterial forward reads at position 290, and the reverse reads at position 250. For eukaryotic sequences, we trimmed both forward and reverse reads at position 250. Because of the low quality of the reverse reads, we only kept the forward reads for archaeal sequences and trimmed them at position 210. Unique amplicon sequence variants (ASVs) were then determined with the inference algorithm as implemented in DADA2 [30]. Afterwards, forward and reverse reads were merged using mergePairs and chimeras were removed with removeBimeraDenovo. Taxonomy was assigned using IDTAXA taxonomic classification approach [31] along with SILVA SSU (v.138) database (Retrieved May 01, 2021, from Latin silva, forest, http://www.arb-silva.de, accessed on 23 December 2021) [32]. The non-classified archaeal and eukaryotic sequences underwent a second classification using a dataset construct based on Liu et al. [33] and Zhou et al. [34] for the 16S rRNA archaeal genes, and on the PR^2^ database (Retrieved 12 May 2021, from https://pr2-database.org/) for the 18S rRNA eukaryotic genes. For further analysis of the microbiome data, contaminant sequences present in controls (drilling fluids, blank DNA kit extraction, and negative sample from the PCR amplifications prior to sequencing) were removed from the output sequence table. Processed sequences were aligned with DECIPHER::alignSeqs and a phylogenetic tree was constructed with FastTree (v.2.1.10) [35].

### 2.8. Statistical Analyses

All statistical analyses were performed with R studio (v.4.0.3) using phyloseq (v.1.34.0) [36], and vegan (v.2.5-7) [37]. In order to adjust for differences in library sizes across samples, we used the median sequencing depth normalization method [38] and only used samples with more than 1000 sequences. Taxonomic α-diversity was estimated with Shannon and Simpson indices while phylogenetic α-diversity was estimated with Faith’s PD index [39] and the mean nearest taxon distance (MNTD) index [40]. The combined use of both metrics allows for a better understanding of assemblage structure, while the use of only one metric could lead to incomplete interpretations and biased conclusions [41]. We also calculated the standardized phylogenetic diversity measure of both indices (SES_PD_ and SES_MNTD_) using the null model “taxa.labels” (999 randomization) in Picante R package (v.1.8.2) [42]. The standardized phylogenetic diversity measure expresses how different the observed phylogenetic diversity value is (in units of standard deviations (sd) from the average (mean) phylogenetic diversity in the randomly generated communities. Positive values indicate phylogenetic evenness (co-occurring sequences more phylogenetically distantly related than expected by chance), while negative values indicate phylogenetic clustering (co-occurring sequences more closely related than expected by chance) [43]. We determined the relationships between abiotic factors and depth, and abiotic factors and microbial α-diversity through Spearman correlation analyses. For β-diversity analysis, sample–sample distances were determined with the Bray–Curtis distance and visualized with non-metric multidimensional scaling (nMDS). A Mantel test was used to determine the effects of abiotic factors on β-diversity of microbial communities. In addition, we used analysis of similarities (ANOSIM) to determine if the communities were significantly different among samples with different sedimentary textures. To estimate the proportion of microbial communities from upper soil horizons (sources) contributing to the formation of the microbial communities in deeper soil layers (sinks), we used fast expectation-maximization microbial source tracking (FEAST) [15]. Finally, we investigated potential biotic interactions between bacterial, archaeal, and eukaryotic communities at the ASV-level. Data were filtered to remove bacterial, archaeal, and eukaryotic ASVs from the surface soil and the groundwater samples. Only samples containing sequences from the three domains and the 15 most abundant ASVs from each domain were kept. Then, Spearman correlations were calculated using the relative abundance of each ASV and visualised in a Heatmap. For all statistical analysis, *p*-value <0.05 was considered statistically significant.

## 3. Results

### 3.1. Variation of Chemical Characteristics with Depth

**Figure 3:**
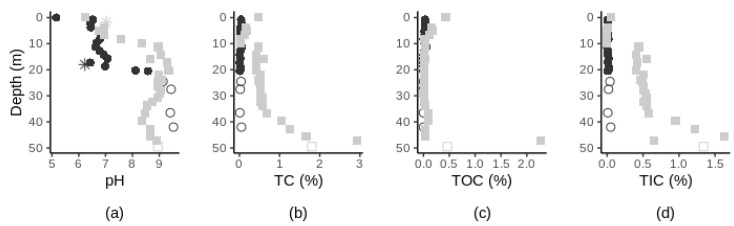
Chemical characteristics of geological material along depth at site 1 (black circles) and site 2 (grey squares) with pH (**a**), TC (**b**), TOC (**c**) and TIC (**d**) along depth. Surface sample at site 1 was removed from the TC, TOC and TIC figures for a better visual representation. Bedrock samples are represented by hollow symbols. pH of the groundwater samples (located at 18 m and 1.75 m of depth, at site 1 and 2 respectively) are indicated by stars in (**a**). For full details on the changes of soil characteristics, see Supplementary Tables S1 and S2.

The chemical characteristics of the geological material (pH, TN, TC, TOC, TIC) varied with depth and between sites (Figure 3). Subsurface pH varied from neutral to basic and significantly increased along soil depth at site 1 (Spearman’s *r* = 0.83, *p*-value < 0.0001, Supplementary Table S3) with the most pronounced changes occurring between depths 20–30 m (site 1) and 0–10 m (site 2) (Figure 3a). The TN percentage values were below our detection limits for all samples except for the near-surface horizons samples (Supplementary Table S1 and S2). The TC, TOC and TIC content varied between sites. At site 1, TC, TOC, and TIC content were higher in the surface soil sample than in the subsurface samples (Supplementary Table S1). In the subsurface samples, the TC content was variable throughout the profile (ranging from 0.01 to 0.06% across all subsurface samples, Figure 3b). TOC content was higher in the top 13 m samples in comparison to deeper samples where % TOC were all below our detection limits. At site 2, TC content in the subsurface significantly increased with depth (Spearman’s *r* = 0.91, *p*-value < 0.0001, Supplementary Table S3). TOC content was variable throughout the soil profile (ranging from 0.01 to 2.27% across all samples), decreasing by over six orders of magnitude from the four shallowest samples to the following twenty samples and finally increased by nearly 45 orders of magnitude in the second deepest samples (Figure 3c). At both sites, % TIC in the subsurface significantly increased with increasing depth (Spearman’s *r* > 0.55, *p*-value < 0.01, Supplementary Table S3) (Figure 3d). Sedimentary texture changed with depth at both study sites. At site 1, as depth increased, soil texture varied continuously from finer materials to coarser materials, while at site 2, soil texture varied generally from coarser materials (e.g., medium sand) at the surface to finer materials (e.g., clay) (Supplementary Table S1 and S2). At site 1, pH, % TC, and % TIC increased in the bedrock samples (Figure 3, hollow circles). At both sites, groundwater pH was close to neutrality (Supplementary Table S1 and S2).

**Figure 4:**
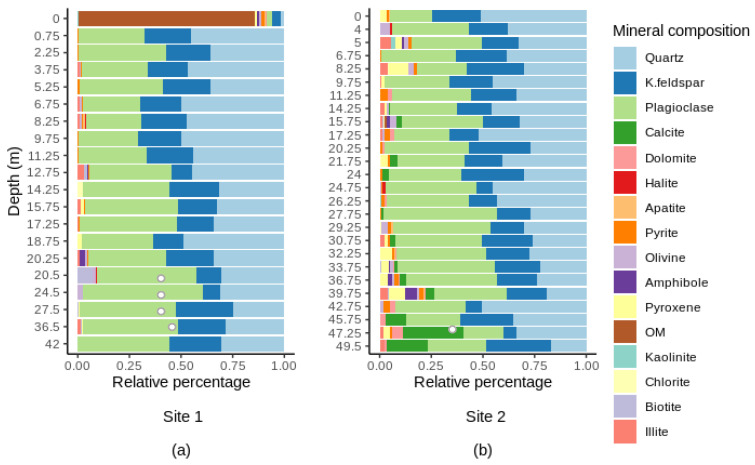
Mineral composition along depth at site 1 (**a**) and at site 2 (**b**). OM, organic matter. Bedrock samples are indicated by hollow symbols. For full details on the mineralogical composition, see Supplementary Tables S4 and S5.

The subsurface samples taken in the scope of this study were characterized by a mosaic of different minerals (Figure 4). They were predominantly composed of quartz (SiO_2_), plagioclase (NaAlSi_3_O_8_–CaAl_2_Si_2_O_8_) and K-feldspar (KAlSi_3_O_8_). The surface sample at site 1 was mostly composed of organic matter (~85%) (Figure 4a). The bottom three samples at site 2 were characterized by the presence of detrital carbonates (calcite and dolomite) which are abundant in rocks from the St. Lawrence Platform (Figure 4b). At both sites, the relative abundance of quartz significantly decreased with depth (Spearman’s *r* < −0.40, *p*-value < 0.05). There were also strong significant relationships between depth and the content of plagioclase (site 1), calcite (CaCO_3_) and dolomite (CaMg(CO_3_)_2_) (site 2) (Spearman’s *r* > 0.60, *p*-value < 0.01, in all cases).

### 3.2. Taxonomic α-Diversity: Variations with Depth and Correlation with Chemical Characteristics of Geological Material

Bacterial taxonomic α-diversity decreased significantly with depth whether diversity was expressed by the Shannon or Simpson indexes (Spearman’s *r* < −0.50, *p*-value < 0.05, Supplementary Table S5 and Supplementary Figure S1) with the most pronounced change occurring when the bedrock was reached, between 20 and 24 m of depth at site 1 (Supplementary Figure S1a,b). We found significant relationships between bacterial taxonomic α-diversity metrics and pH, % TOC, and % TIC at site 1, and with % TC and % TIC at site 2 (Supplementary Table S6). Archaeal taxonomic α-diversity peaked at intermediate depth (~10–15 m) and was lower at the surface and in the deepest horizons (Supplementary Figure S1e,f). The archaeal taxonomic α-diversity significantly decreased along the soil profile (0–46 m) at site 2 (Spearman’s *r* < −0.60, *p*-value < 0.01, Supplementary Table S7). We found negative significant relationships between the archaeal taxonomic α-diversity metrics and % TC, % TIC at site 2 (Spearman’s *r* < −0.55, *p*-value <0.01, Supplementary Table S7). Eukaryotic α-diversity did not show clear trends with depth or any other geological material characteristics (*p*-value > 0.05, in all cases, Supplementary Table S8). However, some of our samples, especially the deepest ones contained a very low number of sequences for archaea and eukaryotes (<1000 reads) and these samples were not used for comparisons of taxonomic α-diversity or any of the subsequent analyses.

### 3.3. Phylogenetic α-Diversity and Correlation with Chemical Characteristics

At site 2, bacterial phylogenetic α-diversity either significantly increased with depth when measured with MNTD index (Spearman’s *r* = 0.80, *p*-value < 0.0001, Supplementary Table S6) or decreased with depth when measured with Faith’s PD (Spearman’s *r* =−0.75, *p*-value < 0.0001, Supplementary Table S6, Supplementary Figure S1c,d). As observed with the taxonomic α-diversity indices, the most pronounced changes in phylogenetic diversity occurred when the bedrock was reached. Using Spearman correlation, we also found significant relationships between the bacterial phylogenetic α-diversity metrics and % TC and % TIC at site 2 (Supplementary Table S6). Archaeal phylogenetic α-diversity changed significantly with depth when measured with Faith’s PD index (Supplementary Table S7) and showed opposite patterns in the studied sites (Supplementary Figure S1g,h). In the depth interval where archaeal sequences were detected at site 1 (0.75–20 m), we found an increase of Faith’s PD with depth (Spearman’s *r* = 0.73, *p*-value < 0.01). By contrast, in the 0–46 m depth interval where archaeal sequences were detected at site 2, the archaeal phylogenetic α-diversity decreased with increasing depth (Spearman’s *r* < −0.73, *p*-value < 0.0001). Faith’s PD was also correlated with % TC (both sites), % TOC (both sites) and % TIC (site 2) (*p*-value < 0.05, in all cases, Supplementary Table S7). Using only samples containing more than 1000 sequences (we did not use 22 samples, including six samples from the site 1 and 16 of the deepest samples at site 2), we did not find a clear relationship between the phylogenetic eukaryotic α-diversity metrics and soil depth at either site (Supplementary Table S8; Supplementary Figure S1k,l). In addition, the majority of the standardized effect sizes of the MNTD (SES_MNTD_) and Faith’s PD (SES_PD_) values obtained using the null model were below zero (SES_MNTD_ < 0 and SES_PD_ < 0), meaning that the sequences in the terrestrial subsurface samples’s were more closely related to each other than expected by chance [43].

### 3.4. β-Diversity and Correlation with Depth and Chemical Characteristics

**Figure 5:**
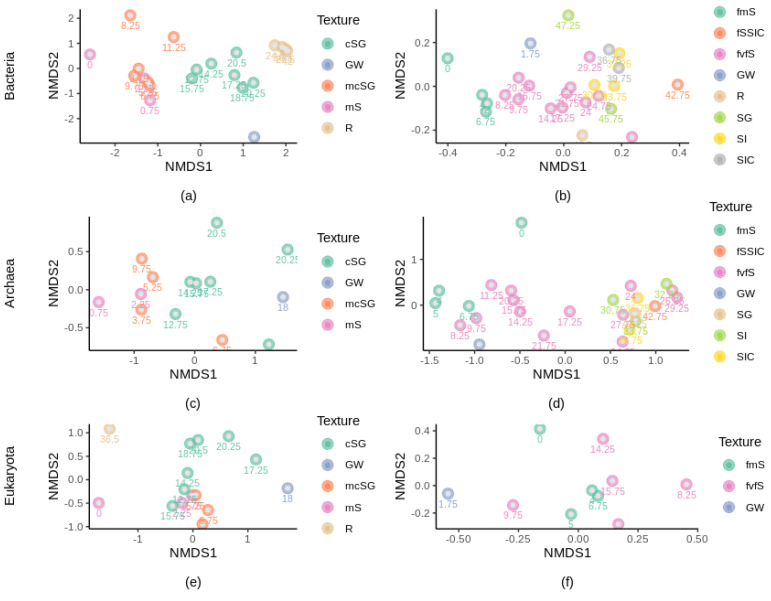
Ordination of microbial communities in the subsurface layers with different textures as indicated by nonmetric multidimensional scaling plots (nMDS) based on Bray–Curtis dissimilarity matrices. Textures are color coded, and depth is indicated for each sample (subscript). With: (**a**) bacterial composition at site 1, stress = 0.113; (**b**), bacterial composition at site 2, stress = 0.159; (**c**) archaeal composition at site 1, stress = 0.0094; (**d**) archaeal composition at site 2, stress = 0.137; (**e**) eukaryotic composition at site 1, stress = 0.0107; (**f**) eukaryotic composition at site 2, stress = 0.133. mS, medium sand; mcSG, medium to coarse sand and gravel; cSG, coarse sand and gravel; R, bedrock; fmS, fine to medium sand; fSSIC, fine sand with silt and clay; fvfS, fine to very fine sand; SG, sand and gravel; SI, silt; SIC, silt and clay; GW, groundwater.

The β-diversity levels (i.e., the Bray–Curtis values) changed significantly with depth, for all microbial domains and at both study sites (*p*-value < 0.05, Supplementary Table S9). At both sites, depth, texture and pH were the major factors shaping bacterial communities (Figure 5, Supplementary Table S9). For the archaeal community, we found moderate correlations between the β-diversity levels and, pH and TC content (Supplementary Table S9). Finally, the eukaryotic community composition was more strongly correlated with % TC (site 1) and % TOC (site 2) rather than with depth (Supplementary Table S9). We did not find any strong correlations between the microbial composition and the mineralogy composition. At both sites and for all microbial domains, the community composing the groundwater samples was distinct from the community in the geological material (Figure 5).

### 3.5. Absolute Bacterial Abundance

Absolute bacterial abundance determined with dPCR was clearly higher in the soil surface in comparison with the subsurface (Table 1). Using only samples with a good quality threshold, we found that the absolute bacterial abundance declined significantly with depth at site 2 (Spearman’s *r* = −0.68, *p*-value < 0.01, Supplementary Table S10). We also found negative correlations of bacterial absolute abundance with % TC and % TIC (site 2, Supplementary Table S10).

### 3.6. Variation in the Relative Abundance of Dominant Bacteria, Archaea, and Eukaryote Microorganisms at the Phylum and Genus Levels, with Depth

**Figure 6:**
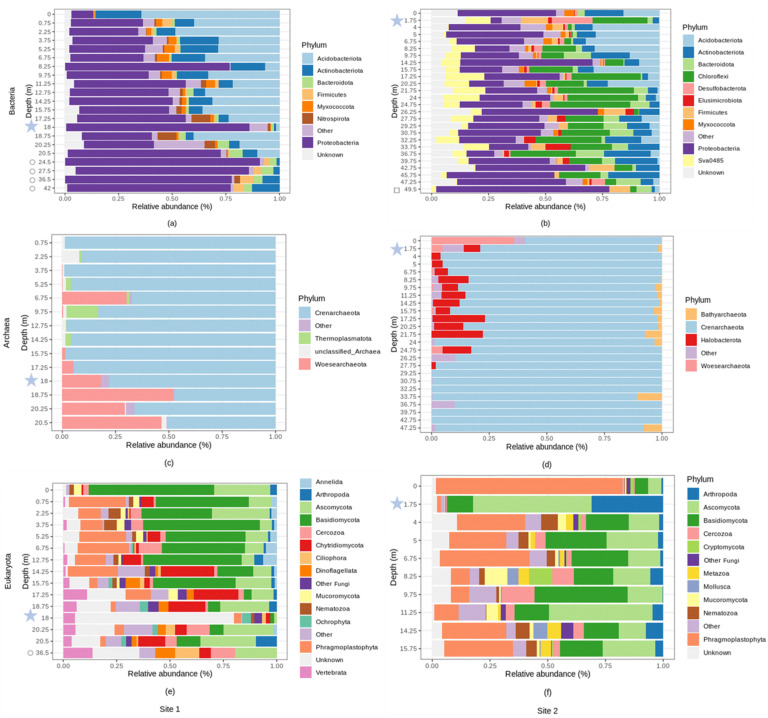
Relative abundance (%) of microbial phyla across different depths (m) at site 1 and at site 2. With: (**a**) bacterial relative abundance at site 1, (**b**) bacterial relative abundance at site 2, (**c**) archaeal relative abundance at site 1, (**d**) archaeal relative abundance at site 2, (**e**) eukaryotic relative abundance at site 1, (**f**) eukaryal relative abundance at site 2. Phyla with an average relative abundance of less than 1% were categorized as “Other”. Bedrock samples are indicated by hollow symbols. Groundwater samples are located at 18 m and 1.75 m of depth, at site 1 and 2, respectively, and are indicated by blue stars.

At site 1, the dominant bacterial phyla belonged to Proteobacteria (51.62% average relative abundance across all samples), Acidobacteriota (23.94%), and Actinobacteriota (9.37%). At site 2, the dominant bacterial phyla were represented by Proteobacteria (32.93%), Chloroflexi (15.60%), and Acidobacteriota (9.64%) (Figure 6a,b). The dominant bacterial genera belonged to Ralstonia (15.90%), and Acinetobacter (2.33%) at site 1, and Acinetobacter (8.30%) at site 2 (Supplementary Figure S2a,b). Their relative abundances were highly variable across the collected samples with the most changes occurring when the bedrock was reached, around 20–25 m of depth at site 1 (Supplementary Figure S2a,b). Relative abundance of the phylum Proteobacteria increased with depth at site 1 (Supplementary Table S11) while relative abundances of Acidobacteriota and Myxococcota declined exponentially with depth at both sites (Supplementary Table S11, Supplementary Figures S3 and S4). Some phyla like Actinobacteria were relatively most abundant at the surface and in the deepest horizons while the opposite pattern was observed for Nitrospirota (site 1, Supplementary Figure S5). Likewise, most of the genera at site 1: *Bryobacter*, Candidatus Koribacter, *Acidothermus* decreased exponentially in relative abundance and were not found in the deepest samples (Supplementary Table S11, Supplementary Figure S5). The opposite pattern was observed for *Ralstonia,* the relative abundance of which increased with depth (Supplementary Table S11) and peaked between 20–25 m of depth where this genus comprised up to 50% of the community (Supplementary Figure S5). The relative abundances of the other most abundant phyla and genera did not exhibit any clear shifts with depth (Supplementary Table S11, Supplementary Figure S6). Sessile/endolithic and planktonic communities shared similar taxa which however differed in their relative abundances (Figure 6). Sessile/endolithic bacterial communities surrounding the groundwater contained more Acidobacteria than the planktonic communities (Figure 6a,b). At site 2, there were more Chloroflexi (23.97% in the groundwater vs. 0.06% and 1.56% in the surrounding solid materials), sva0485 (11.15% vs. 0.00% and 0.56%), Firmicutes (11.81% vs. 0.12% and 4.87%), Desulfobacterota (17.98% vs. 1.15% and 1.30%), and less Myxoccocota (0.08% vs. 4.281% and 2.71%) in the groundwater (Figure 6a,b). In addition, some abundant bacterial phyla were solely detected in the geological material and not in the groundwater (e.g., *Ralstonia, Acinetobacter, Bryobacter, Rhodoplanes* and Candidatus Koribacter, site 1, Figure 6a).

At both sites, the archaeal communities were dominated by the Crenarchaeota phylum which comprised up to 80% of the total community (Figure 6c,d). At site 1, the archaeal community was also represented by Woesearchaeota (13.10%) and Thermoplasmatota (1.62%). Nearly all the Crenarchaeota sequences (%) were classified as members of group 1.1c (54.43%, Supplementary Figure S2c), which was dominant in the surface soil sample, and decreased with depth (Supplementary Table S11, Figure S7). At site 1, the Woesearchaeota subgroup 24 increased in relative abundance with depth (Supplementary Table S11, Supplementary Figures S2c and S7)**.** At site 2, unclassified Bathyarchaeia, Crenarchaeota and Group 1.1c were dominant (59.77, 23.39 and 3.32% respectively). The relative abundance of unclassified Crenarchaeota and unclassified Group 1.1c decreased with depth, whereas unclassified Bathyarchaeia increased (Supplementary Table S11, Supplementary Figure S8). Methanogens such as Methanosarcina, Methanoregula or the uncultured Rice Cluster II, and the methanotroph cand. Methanoperendens were detected until 20 m depth and were also in the groundwater (Supplementary Figure S8). The Marine Group II was detected in a vast majority in the groundwater.

At site 1, the most abundant eukaryotic phyla belonged to Basidiomycota (26.42%), Ascomycota (14.87%), Phragmoplastophyta (10.64%), Chytridiomycota (7.65%), Cercozoa (4.24%), Vertebrata (3.70%), Dinoflagellata (2.06%) and Arthropoda (1.92%) (Figure 6e). At site 2, the most abundant eucaryal phyla were represented by Phragmoplastophyta (25.99%), Ascomycota (21.69%), Basidiomycota (19.20%), Arthropoda (5.88%), Cercozoa (4.47%), Nematozoa (2.99%), Mucoromycota (2.51%) and Metazoa (2.37%) (Figure 6f). Some of these phyla (e.g., Basidiomycota, Nematozoa, Vertebrata, Dinoflagellata, Mucoromycota) changed significantly in relative abundance with soil depth at site 1 (Supplementary Table S11). The most abundant eukaryotic genera at site 1 belonged to Geranomyces (~4.42%), Archaeorhizomyces (~1.69%) and Oikomonas (~1.05%, only detected in the groundwater) while the most abundant eukaryote genera at site 2 were Venturia (~1.97%) and Archaeorhizomyces (~1.66%) (Supplementary Figure S2e,f). The unidentified genera (referred to as the unknown genera) accounted for up to 80% of the average relative abundance across all samples analyzed. At site 2, we found less Phragmophyta (1.73% vs. 80.92% and 29.53%), more Ascomycota (51.12% vs. 5.83% and 13.18%), and more Arthropoda (31.11% vs. 0.82% and 1.83%) in the groundwater than in the geological material. In addition, some abundant eukaryotic taxa were solely detected in the rocks and geological material and not in the groundwater (e.g., Geranomyces, site 1 and Archaeorhizomyces, site 2).

### 3.7. Interactions among Bacterial, Eukaryotic and Archaeal major ASVs

**Figure 7:**
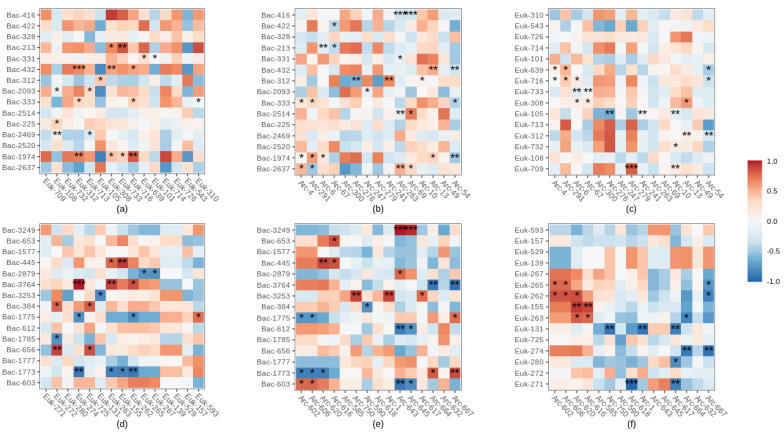
Spearman correlations between dominant bacterial, archaeal and eukaryotic ASVs. With correlations between bacterial and eukaryotic ASVs at site 1 (**a**) and site 2 (**d**), bacterial and archaeal ASVs at site 1 (**b**) and site 2 (**e**), eukaryotic and archaeal ASVs at site 1 (**c**) and site 2 (**f**). Both positive (blue) and negative (red) correlations between ASVs were calculated using the relative abundance of dominant ASV. Data were filtered to remove ASVs from the surface soil and the groundwater samples. Significant correlations are noted with a * (when *p* < 0.05), ** (*p* < 0.01) or *** (*p* < 0.001).

There were significant positive, negative and neutral correlations between the most abundant ASVs of the three domains (Figure 7). Most of these interactions were positive, between bacterial and eukaryotic ASVs (especially at site 1) while they were mostly negative between archaeal and eukaryotic dominant ASVs at site 2 (Figure 7f). Bacterial and eukaryotic interactions prediminantly occurred between ASVs belonging to the phylum Phragmoplastophyta (e.g., Euk312 at site 1 or Euk-274 or/and Euk-263 at site 2) or the phylum Basidiomycota (e.g., ASV-Euk 716 at site 1) (Figure 7a,d). Bacterial ASV Bac-1974 (site 1) and 3764 (site 2) belonging to the Xanthobacteraceaea family were strongly positively correlated with most of the eukaryotic ASVs (Figure 7a,d). In contrast, Acinetobacter spp. (Bac-2469 at site 1, Bac-1775, Bac-1785, Bac-1773 at site 2) were negatively correlated with most eukaryotic ASVs (Figure 7a,d). Most archaeal interactions occurred with the Crenarchaeota Group 1.1c at site 1 (e.g., Arc-4, Arc-291, Arc-6, Arc-276) or with unclassified Crenarchaeota at site 2 (Figure 7d,e). These taxa correlated both positively and negatively with dominant bacteria. At site 1, eukaryotic ASV 105 (phylum Chytridiomycota), was strongly negatively correlated with most of the archaeal ASVs (Figure 7c).

### 3.8. Microbial Source Tracking

**Figure 8:**
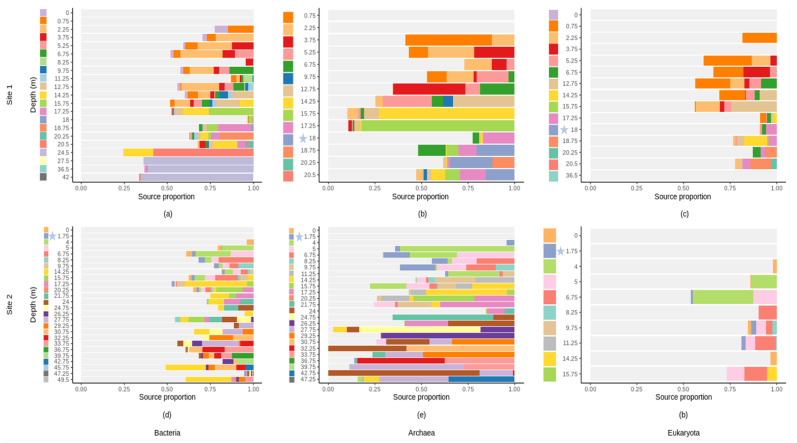
FEAST estimations of the proportion of communities from upper layers (source) contributing to the formation of communities in the deeper layer (sink) across different soil depths (m) at site 1 and at site 2. (**a**,**d**) for bacterial communities; (**b**,**e**) for archaeal communities; (**c**,**f**) for eukaryotic communities. The unknown sources are indicated in grey. Each color indicates the sources which are the microbial communities from each sample (e.g., in (**a**), the sample collected at depth 2.25 m has sources which are the microbial communities from the upper layers (the surface soil at depth 0 m (purple) and the soil layer at depth 0.75 m (orange)). Groundwater samples are located at 18 m and 1.75 m of depth, at site 1 and 2, respectively, and are indicated by blue stars.

According to the FEAST analyses, upper samples contribute to the formation of shallow subsurface bacterial, archaeal and eukaryotic communities (Figure 8). They contribute on average to 48.18% (site 1) and 61.56% (percentage of the contribution of upper soil horizons) (site 2) for the formation of archeal communities in the profile (Figure 8b,e); 35.91% (site 1) and 26.26% (site 2) for the bacterial communities (Figure 8a,d); and 19.14% (site 1) and 13.66% (site 2) for the eukaryotic communities (Figure 8c,f). The remaining proportions were due to “unknown sources”. The contribution of the groundwater community to the geological material/rock communities was extremely low, especially for bacterial and eukaryotic communities (either nonexistent or below 0.001%) (Figure 8).

## 4. Discussion

### 4.1. The Effect of Depth on Endolithic and Sessile Communities

Our study showed that both bacterial taxonomic α-diversity and absolute abundance decreased with soil depth. Archaeal taxonomic α-diversity followed the same pattern, while eukaryotic diversity did not differ significantly between soil depths at both sites. The decrease in bacterial taxonomic α-diversity with depth is commonly described [5,7,8,9,10,11,12,14,44,45,46,47], whereas studies investigating the vertical pattern of Archaea tend to show no consistent conclusion [6,44,47,48]. However, these studies only focused on the first centimeters or meters of subsurface soils. Our result suggests that changes in abiotic factors with depth represent a strong ecological filter [46]. Therefore, most Bacteria and Archaea that live in the shallow terrestrial subsurface are less likely to thrive in deeper environments [44]. We associate our nonsignificant results for eukaryotic taxonomic α-diversity with the fact that we were not able to analyze the deeper samples due to very low sequence numbers. As supported by our inability to measure absolute abundances via digital PCR, this may be an indication that the eukaryotic microbial population is very low/absent in the deepest samples. In these deeper subsurface horizons, eukaryotes are probably limited in space, nutrients, and are unable to cope with oxygen limitations, or some combination thereof [49].

The variation in phylogenetic α-diversity patterns of microbial communities along soil depths showed contrasting results depending on the targeted microbial domains, the study site, and the index used for α-diversity measuring. Bacterial phylogenetic α-diversity along the profile tended to decrease when measured with Faith’s PD index but increased with MNTD. For Archaea, only Faith’s PD was correlated with depth, but patterns were opposite in the studied sites (an increase in the first study site (~0.75–20 m) and a decrease in the second study site (~0–47 m)). In addition, eukaryotic phylogenetic α-diversity did not show any clear trend with depth. Opposing patterns of phylogenetic diversity metrics might simply reflect differences in metric properties [41]. The index PD estimates the phylogenetic diversity of a community as the sum of the tree branch lengths connecting all species [50]. As a result, Faith’s PD and species richness are also often highly correlated [51]. This fact probably explains the behaviour of this index. The MNTD index is the average phylogenetic distance between any taxon and its closest relative [50]. This measure of phylogenetic diversity increased for bacteria in the deeper horizons. Similar results were observed by Chu et al., and this difference was linked to an increased importance of environmental filtering in surface soils in comparison to deep horizons [1]. However, based on our results regarding the association between chemical characteristics and taxonomic α-diversity, we consider this unlikely to be the case here. Coexistence of phylogenetically distant bacteria in stressful and resource-poor sites—like in the deeper horizons of the shallow terrestrial subsurface—may suggest that competition between close relatives is exerting great selection pressure. Together with taxonomic diversity result, these results suggest that a small number (that is, low taxonomic α-diversity and low absolute abundance) of phylogenetically distant bacterial taxa can or must co-exist to survive the stressful conditions of the deeper horizons of the shallow terrestrial subsurface. In oligotrophic habitats, lack of nutrients can lead to a reduction in genome size by loss of expendable genes [52]. Taken to extremes, this can lead to the loss of essential metabolic functions, which inevitably leads to dependencies on other possibly phylogenetically distant organisms [52]. The fact that MNTD index showed no correlation with depth for both archaea and eukaryotes suggest no contributions of history and trait evolution to this community structure [53]. This result may suggest that (within the depth interval considered), archaeal and eukaryotic community membership is not limited by a requirement for shared stress tolerance traits, or that there are no dependencies on other phylogenetically distant organisms, or no strong competition between phylogenetically close organisms [53]. In addition, the majority of the standardized effect sizes of MNTD and Faith’s values obtained for bacteria, archaea and eukaryotes using the null model were below zero, which shows that the microbial communities tended to be more phylogenetically clustered than would be expected by chance [43]. Therefore, in agreement with our taxonomic α-diversity results, microbial communities from the shallow terrestrial subsurface are structured by environmental filtering [1].

### 4.2. Effect of Abiotic Characteristics on Endolithic and Sessile Communities

The studied profiles from both sites (0–50 m) represent strong environmental gradients, with multiple abiotic factors changing with depth. One of the most pronounced changes through the profiles was the increase in pH with depth (toward more basic pH) and the relatively low nitrogen and carbon organic quantity. In the studied sites, sedimentary texture does not seem to be a major factor influencing the α-diversity patterns as opposite sedimentary texture patterns (coarser and looser at site 1 vs. finer and denser at site 2) are not reflected by opposite or different α-diversity patterns. Yet, soil texture can affect microbial movement through soils and finer soils, especially clay minerals, increase water and nutrient retention which should increase survival time [54,55]. Hence, if sedimentary texture was the predominant factor influencing microbial communities, the deeper and finer soil horizons at site 2 should have contained a higher diversity than the shallower and coarser soil horizons. Our results suggest that other factors are predominant in controlling the α-diversity of the subsurface microorganisms in the studied sites. We found significant relationships of bacterial absolute abundance and some of the bacterial and archaeal α-diversity metrics with abiotic factors such as pH, % TOC, % TIC, or/and % TC. Bacterial and archaeal taxonomic α-diversity tended to show a negative correlation with the concentration of TIC. This suggests that bacteria and archaea from the shallow terrestrial subsurface mostly acquire energy from organic carbon. However, because all these variables are moderately to strongly correlated with depth, we do not know if the changes induced by these variables are the sole factor driving the observed α-diversity patterns. Soil microbial communities may also be directly or indirectly affected by other factors, such as water content, oxygen quantity, trophic status, spatial and climate factors [12,48]. 

The bacterial, archaeal, and eukaryotic community compositions showed differences with depth through the whole profiles and mineral composition does not appear to play a role in the structure of microbial communities. In accordance with our results, it has been reported that soil microbial communities are strongly shaped by soil properties such as nutrient availability, pH, and soil texture, which vary considerably with soil depth [12,56,57]. Changes in the composition of the bacterial communities with pH, especially at site 1, is not surprising given the predominance of members of the Acidobacteria phylum (e.g., *Candidatus Koribacter, Acidothermus, Bryobacter*). Although Acidobacteria are generally oligotrophic and might be more adapted to the environment in deep layers [57], there is some evidence to suggest that their abundance within a community is primarily regulated by pH [58].

The overall structure of the bacterial communities also changed markedly with geological material texture, with the most pronounced changes occurring within the bedrock (site 1). The taxonomic α-diversity in site 1 also suddenly decreased at the level of the bedrock. Together, these results suggest that the conditions in the bedrock changed markedly and that many surface-dwelling bacteria are less likely to thrive in this environment. We associate this trend with the little space for water and microbe colonization that the bedrock usually provides [49]. The increase in relative abundance of *Ralstonia,* a member of Proteobacteria in this environment is probably due to their capabilities to cope with hostile life conditions, such as extreme pH or oligotrophic environments [59]. As reported by many previous studies, ammonia-oxidizing archaea dominated the profiles (e.g., 12, 48), which may drive autotrophic nitrification in the deeper depths [12,48]. The relative abundance of the Crenarchaeote phylum members decreased at site 1 but increased at site 2. Their decrease might be explained by the presence of group 1.1c at the first study site, a group that prefers acidic soils and has even been detected at a pH below 3 [48]. This different trend suggests that changes in microbial community composition with depth are, to some degree, site specific and dependent on specific characteristics of the profiles being studied. At site 2, H_2_/CO_2_-utilizing autotrophic methanogens were identified (*Methanoregula*, *Methanosarcina*, Rice Cluster II), possibly linked to the higher TIC content at this site. The presence of the methanotroph cand. Methanoperedens suggests a coupling between methane production, and the nitrogen cycle [60,61,62].

The eukaryotic community composition was not primarily controlled by depth. Instead, carbon content best explained the observed patterns. Predominant eukaryotic taxa at both study sites were mainly assigned to Basidiomycetes and Ascomycetes phyla, a result also observed by Hartmann et al. [2] and Xu et al. [14]. The eukaryotic community might be controlled by other abiotic factors such as soil moisture [63], or by vegetation types as suggested by Xu et al. [14]. Plant communities may play a major role in structuring eukaryotic microbial community composition, particularly for eukaryotic communities dominated by fungi [64]. The decrease of the relative abundance of Basidiomycota can probably be explained by the fact that the representatives of these fungi can form symbiotic relationship with plants [14]. However, the decrease of Basidiomycota was only detected at site 1 and other studies have shown an inconsistent pattern of fungal composition along soil depths [65]. The inconsistent pattern may suggest that site-specific characteristics are also important determinants for shaping the fungal communities. Further studies on the ecology of these diverse eukaryotic taxa in the shallow terrestrial subsurface are clearly needed [66].

The detection of Vertebrata, Metazoa and Nematozoa in the geological material in deep soil horizons is surprising even if multicellular life in the deep subsurface has been previously detected in this habitat [67]. The presence of these organisms in the terrestrial subsurface can mean that the material examined contained the remains of the above animals passively infiltrating through the depth. Overall, this result highlights the importance of the surface community’s role in structuring the diversity and composition of subsurface communities as many other organisms can passively be brought from the surface to the terrestrial subsurface (e.g., plants, fungi).

### 4.3. Potential Biotic Interactions between Bacterial, Eukaryotic and Archaeal Microorganisms

Soil microorganisms coexist in complex associations, including mutualism, competition, predation or neutral [68]. In the shallow terrestrial subsurface, we found that most of the correlations between bacterial–eukaryotic microorganisms were positives while a lot of correlations between archaeal–eukaryotic microorganisms were negatives (especially at site 2). Positive correlations may indicate the occupation of different niches [69], cooperative or mutualistic potential associations such as cross-feeding and/or syntrophic relationships [68]. Eukaryotic organisms are unable to obtain fixed nitrogen without nitrogen-fixing prokaryotes [70]. Hence, positive interactions detected between eukaryotic organisms and nitrogen-fixing bacteria, such as the Xanthobacteraceae family (e.g., ASV Bac-1974 at site 1 and Bac-3764 at site 2) suggest symbiotic interactions between these two domains [70]. Negative correlations could reveal antagonistic associations among species, such as competition for limited resources between archaeal and eukaryotic microorganisms, but it could also point out to predation and/or parasitism interactions [70]. Some chytrids, for example are host-specific parasitic fungi, and may have a considerable negative impact on archaeal cells [71]. Our data contained a large number of unknown taxa at the genus-level. Precise identification is critical to gain more precision on the nature of the biotic interactions between the three domains. We also did not take into account the biotic interactions within each domain (e.g., bacterial–bacterial), but negative, positive and neutral correlations can also occur within domains and have a big influence on the whole community’s structure.

### 4.4. Vertical Fluid Fluxes as Sources of Microbial Communities in the Shallow Terrestrial Subsurface

A major challenge of analyzing the compositional structure of microbial communities is to identify their potential origins and sources. The composition of each microbial community is typically composed of the members of several environmental sources, including different contaminants as well as other microbial communities that interact with the sampled habitat [15]. In the shallow subsurface, members of the microbial communities can be long-term descendants of microorganisms that colonized the geological material during deposition [72] or represent younger/recent surface-sourced colonists or temporal survivors that disperse by diffusion, or by vertical and lateral fluid fluxes [3,13,72]. Our objective was to estimate the proportion of shallow subsurface microbial communities that originated through vertical fluid fluxes by estimating the proportion of microbial communities from upper horizons (sources) contributing to the formation of the microbial communities in deeper soil layers (sinks). Our FEAST analysis confirmed a vertical microbial colonization of the geological material and of the bedrock in the shallow terrestrial subsurface as upper horizon communities were sources for deeper layers (sinks).

The higher contribution of upper samples to the formation of archaeal communities might be explained by their better tolerance to extreme environments [14], which allow better survival in deeper layers from surface colonization. Downward fluid fluxes might be an additional explanation for the decrease in bacterial and archaeal taxonomic α-diversity and/or absolute abundance with depth. Indeed, microorganisms arriving in deeper horizons from the surface are more likely adapted to live under the surface or near surface conditions. However, the other microbial sources, collectively referred to as the “unknown source” still represent an important proportion of potential sources, especially for subsurface eukaryotic communities. Other potential sources might come from plants, or rainwater and more work is clearly needed (especially for eukaryotes) to unravel the origin of these complex subsurface microbial communities.

Another potential source of microbial cells might be the community found in the groundwater. However, the FEAST analyses suggest little to no contribution from planktonic populations to the overall sessile/endolithic community. Both communities have different compositions despite the presence of similar taxa which might suggest some exchange between the two communities. On the other hand, some microbial taxa were exclusively found either in the groundwater or in the surrounding geological material and bedrock. This is the case with the heterotrophic nanoflagellate *Oikomonas*, or the potential nitrate-reducing Marine Group II archaea, who show a preference for the planktonic form as they were found solely in the groundwater. In addition, differences in composition can partly be explained by the differences in abiotic factors in water compared to surrounding geological material and bedrock [73,74]. For example, the pH is closer to neutrality in the groundwater in comparison to the surrounding geological material and bedrock (Supplementary Tables S1 and S2). However, other characteristics could also affect these communities (e.g., connectivity, nutrient inputs, etc.). Our results do not allow us to conclude whether rock-associated communities are significantly different from their planktonic counterparts. However, the bar plot (Figure 6) and the nMDS analysis allowed us to observe marked differences in the composition of these two communities and other authors such as Lazar et al. [3] suggested the existence of a unique rock matrix microbiome compared to the surrounding groundwater. In accordance with Griebler et al. [75], our results underline the importance of sampling both the attached and the suspended communities when studies on all the communities of the aquifer are performed. Studies and predictions on the functioning of subsurface ecosystems based solely on groundwater samples might not include some taxa (although they might be rare) and therefore might be subjected to misinterpretation [3]. A comparative study with a larger number of subsurface water samples and surrounding rocks is needed to draw robust conclusions about the existence of different communities in the aquifer (planktonic vs. rock-associated microbiome).

## 5. Conclusions

In the studied sites, many abiotic factors changed with depth (including soil texture, pH, carbon content, nitrogen content and mineralogy). Depth can therefore be seen as an ecological and phylogenetic filter for the subsurface microbial communities. Thus, most bacteria, archaea (and probably eukaryotes, although our data cannot confirm) are less likely to thrive in the deeper horizons. In addition, the significant effect of depth could be explained by vertical flow movements in the subsurface layers. Our results suggest that these vertical movements supply the earth’s subsurface with microorganisms. It is therefore very likely that these communities—which are transported from the surface to the deeper soil horizons—are less adapted to the conditions of the deeper horizons and may use various survival strategies. We found that, in the deeper horizons, the competition is strong for the phylogenetically close taxa and that therefore, the cohabitation of a reduced number of phylogenetically distant bacterial taxa is an advantage (different ecological niche/form of co-dependency). Our results also suggest that cooperative or mutualistic potential associations between bacteria and microbial eukaryotes occur, such as cross-feeding and/or syntrophic relationships in the terrestrial subsurface. 

The high heterogeneity of soil makes it difficult to achieve a systematic understanding of the microbial community distribution in subsurface soils across large-scale regions [48]. To get a more comprehensive insight into the distribution and biogeography, particular attention should be paid to eukaryotic communities, and further studies using samples from different habitats and at a larger spatial scale are needed in the future [48]. We believe that this study represents a step toward an insightful understanding of the overall assemblage processes affecting microbial communities in the shallow terrestrial subsurface.

## Figures and Tables

**Table 1:** Absolute abundances expressed in number of bacterial 16S rRNA gene copies per gram of geological material (copies g^−1^) detected in each sample. Only values with good quality thresholds are presented.

Site	Samples	Depth (m)	Abundance (Copies g^−1^)
Site 1	RR-01	0	682,273,937.5
	RR-02	0.75	109,185
	RR-03	2.25	3,618,757.75
	RR-04	3.75	252,068
	RR-05	5.25	344,150.25
	RR-06	6.75	440,495.5
	RR-11	14.25	203,594.5
	RR-12	15.75	569,690.5
	RR-14	18.75	475,723.25
	RR-16	20.5	18,367,280.5
Site 2	NDDL-01	0	539,684,562.5
	NDDL-02	4	222,415.5
	NDDL-03	5	190,000.75
	NDDL-04	6.75	273854.25
	NDDL-05	8.25	83,918.75
	NDDL-06	9.75	520,608
	NDDL-07	11.25	365,820.5
	NDDL-08	14.25	367,227
	NDDL-09	15.75	432,781.5
	NDDL-10	17.25	145,616.25
	NDDL-11	20.25	89,283.75
	NDDL-12	21.75	34,321.5
	NDDL-13	24	498,270.75
	NDDL-14	24.75	35,184.25
	NDDL-17	29.25	60,160.5
	NDDL-20	33.75	33,647.25
	NDDL-21	36.75	18,212
	NDDL-22	39.75	97,846
	NDDL-25	47.25	20,474

Samples have been named according to the site’s location and the order of sampling. RR: Riviere- Rouge (Site 1), NDDL: Notre-Dame-du-Laus (Site 2).

## Data Availability

The obtained sequences were deposited in the National Center for Biotechnology Information (NCBI) under the BioProject ID: PRJNA758373.

## Supplementary Materials

The following are available online at https://www.mdpi.com/article/10.3390/microorganisms10010129/s1, Figure S1: Bacterial, archaeal and eukaryotic α-diversity indexes along depth (m) at site 1 (black, circle) and site 2 (grey, square). With: (a,e,i) Shannon; (b,f,j) Simpson index; (c,g,k) Faith’s PD and (d,h,l) MNTD index. Bedrock samples are represented by hollow symbols. Groundwater samples are represented by stars. Figure S2: Relative abundance (%) of each microbial genera across different depths (m) at site 1 and at site 2. With: (a) bacterial relative abundance at site 1, (b) bacterial relative abundance at site 2, (c) archaeal relative abundance at site 1, (d) archaeal relative abundance at site 2, (e) eukaryotic relative abundance at site 1, (f) eukaryotic relative abundance at site 2. Genera with an average relative abundance of less than 1% were categorized as “Other”. Bedrock samples are represented by hollow circles and hollow squares. Groundwater samples are represented by blue stars. Figure S3: Change in the relative abundance of most abundant bacterial phyla with depth at site 1. Figure S4: Change in the relative abundance of most abundant bacterial phyla with depth at site 2. Figure S5: Change in the relative abundance of most abundant bacterial genera with depth at site 1. Figure S6: Change in the relative abundance of most abundant bacterial genera with depth at site 2. Figure S7: Change in the relative abundance of most abundant archaeal genera with depth at site 1. Unc: uncultured. Figure S8: Change in the relative abundance of most abundant archaeal genera with depth at site 2. Unc. B: uncultured Bathyarchaeia. Table S1: Abiotic characteristics measured along depth gradient at site 1. Table S2: Abiotic characteristics measured along depth gradient at site 2. Table S3: The correlations (r) determined by Spearman correlation between abiotic characteristics and depth at site 1 and site 2. Table S4: XRD results showing the mineralogical composition (in %) of the samples collected at site 1. Table S5: XRD results showing the mineralogical composition (in %) of the samples collected at site 2. Table S6: Spearman correlation between bacterial α-diversity metrics and characteristics of geological material at site 1 and site 2. Table S7: Spearman correlation between archaeal α-diversity metrics and characteristics of geological material at site 1 and site 2. Table S8: Spearman correlation between eukaryotic α-diversity metrics and characteristics of geological material at site 1 and site 2. Table S9: Mantel and ANOSIM test between bacteria β-diversity metrics and characteristics of geological material. Table S10: Spearman correlation between absolute abundance and characteristics of geological material at site 1 and site 2. Table S11: Spearman correlation between relative abundances of most abundant bacterial, archaeal, and eukaryotic phyla and genera with characteristics of geological material at site 1 and site 2.

## References

[1] Chu H., Sun H., Tripathi B.M., Adams J.M., Huang R., Zhang Y., Shi Y. (2016). Bacterial community dissimilarity between the surface and subsurface soils equals horizontal differences over several kilometers in the western Tibetan Plateau. Environ. Microbiol..

[2] Hartmann M., Lee S., Hallam S.J., Mohn W.W. (2009). Bacterial, archaeal and eukaryal community structures throughout soil horizons of harvested and naturally disturbed forest stands. Environ. Microbiol..

[3] Lazar C.S., Lehmann R., Stoll W., Rosenberger J., Totsche K.U., Küsel K. (2019). The endolithic bacterial diversity of shallow bedrock ecosystems. Sci. Total Environ..

[4] Flynn T.M., Sanford R.A., Ryu H., Bethke C.M., Levine A.D., Ashbolt N.J., Santo Domingo J.W. (2013). Functional microbial diversity explains groundwater chemistry in a pristine aquifer. BMC Microbiol..

[5] Kim H.M., Lee M.J., Jung J.Y., Hwang C.Y., Kim M., Ro H.M., Chun J., Lee Y.K. (2016). Vertical distribution of bacterial community is associated with the degree of soil organic matter decomposition in the active layer of moist acidic tundra. J. Microbiol..

[6] Eilers K.G., Debenport S., Anderson S., Fierer N. (2012). Digging deeper to find unique microbial communities: The strong effect of depth on the structure of bacterial and archaeal communities in soil. Soil Biol. Biochem..

[7] Fierer N., Schimel J.P., Holden P.A. (2003). Variations in microbial community composition through two soil depth profiles. Soil Biol. Biochem..

[8] LaMontagne M.G., Schimel J.P., Holden P.A. (2003). Comparison of subsurface and surface soil bacterial communities in California grassland as assessed by terminal restriction fragment length polymorphisms of PCR-amplified 16S rRNA genes. Microbial Ecol..

[9] Agnelli A., Ascher J., Corti G., Ceccherini M.T., Nannipieri P., Pietramellara G. (2004). Distribution of microbial communities in a forest soil profile investigated by microbial biomass, soil respiration and DGGE of total and extracellular DNA. Soil Biol. Biochem..

[10] Goberna M., Insam H., Klammer S., Pascual J.A., Sanchez J. (2005). Microbial community structure at different depths in disturbed and undisturbed semiarid Mediterranean forest soils. Microbial Ecol..

[11] Will C., Thürmer A., Wollherr A., Nacke H., Herold N., Schrumpf M., Gutknecht J., Wubet T., Buscot F., Daniel R. (2010). Horizon-specific bacterial community composition of German grassland soils, as revealed by pyrosequencing-based analysis of 16S rRNA genes. Appl. Environ. Microbiol..

[12] Hansel C.M., Fendorf S., Jardine P.M., Francis C.A. (2008). Changes in bacterial and archaeal community structure and functional diversity along a geochemically variable soil profile. Appl. Environ. Microbiol..

[13] Amy P.S., Haldeman D.L., Ringelberg D., Hall D.H., Russell C. (1992). Comparison of identification systems for classification of bacteria isolated from water and endolithic habitats within the deep subsurface. Appl. Environ. Microbiol..

[14] Xu T., Chen X., Hou Y., Zhu B. (2021). Changes in microbial biomass, community composition and diversity, and functioning with soil depth in two alpine ecosystems on the Tibetan plateau. Plant Soil..

[15] Shenhav L., Thompson M., Joseph T.A., Briscoe L., Furman O., Bogumil D., Mizrahi I., Pe’er I., Halperin E. (2019). FEAST: Fast expectation-maximization for microbial source tracking. Nat. Methods.

[16] (2021). Government of Canada. Canadian Climate Normals 1981–2010 Station Data. Environ. Resour..

[17] (2021). Government of Canada. Canadian Climate Normals 1981–2010 Station Data. Environ. Resour..

[18] Ritchey E.L., McGrath J.M., Gehring D. (2015). Determining soil texture by feel. Agric. Nat. Resour. Publ..

[19] Eckert D., Sims J.T. (1995). Recommended soil pH and lime requirement tests. Recommended Soil Testing Procedures for the Northeastern United States.

[20] Butler B., Hillier S. (2021). powdR: An R package for quantitative mineralogy using full pattern summation of X-ray powder diffraction data. Comput. Geosci..

[21] Lazar C.S., Stoll W., Lehmann R., Herrmann M., Schwab V.F., Akob D.M., Küsel K. (2017). Archaeal diversity and CO_2_ fixers in carbonate-/siliciclastic-rock groundwater ecosystems. Archaea.

[22] Alain K., Callac N., Ciobanu M.C., Reynaud Y., Duthoit F., Jebbar M. (2011). DNA extractions from deep subseafloor sediments: Novel cryogenic-mill-based procedure and comparison to existing protocols. J. Microbiol. Methods..

[23] Direito S.O., Marees A., Röling W.F. (2012). Sensitive life detection strategies for low-biomass environments: Optimizing extraction of nucleic acids adsorbing to terrestrial and Mars analogue minerals. FEMS Microbiol. Ecol..

[24] Muyzer G., De Waal E.C., Uitterlinden A.G. (1993). Profiling of complex microbial populations by denaturing gradient gel electrophoresis analysis of polymerase chain reaction-amplified genes coding for 16S rRNA. Appl. Environ. Microbiol..

[25] Klindworth A., Pruesse E., Schweer T., Peplies J., Quast C., Horn M., Glöckner F.O. (2013). Evaluation of general 16S ribosomal RNA gene PCR primers for classical and next-generation sequencing-based diversity studies. Nucleic Acids Res..

[26] Baker G.C., Smith J.J., Cowan D.A. (2003). Review and re-analysis of domain-specific 16S primers. J. Microbiol. Methods.

[27] DeLong E.F. (1992). Archaea in coastal marine environments. Proc. Natl. Acad. Sci. USA.

[28] Gast R.J., Dennett M.R., Caron D.A. (2004). Characterization of protistan assemblages in the Ross Sea, Antarctica, by denaturing gradient gel electrophoresis. Appl. Environ. Microbiol..

[29] Van de Peer Y., De Rijk P., Wuyts J., Winkelmans T., De Wachter R. (2000). The European small subunit ribosomal RNA database. Nucleic Acids Res..

[30] Callahan B.J., Sankaran K., Fukuyama J.A., McMurdie P.J., Holmes S.P. (2016). Bioconductor workflow for microbiome data analysis: From raw reads to community analyses. F1000Research.

[31] Murali A., Bhargava A., Wright E.S. (2018). IDTAXA: A novel approach for accurate taxonomic classification of microbiome sequences. Microbiome.

[32] Quast C., Pruesse E., Yilmaz P., Gerken J., Schweer T., Yarza P., Pelplies J., Glöckner F.O. (2012). The SILVA ribosomal RNA gene database project: Improved data processing and web-based tools. Nucleic Acids Res..

[33] Liu X., Li M., Castelle C.J., Probst A.J., Zhou Z., Pan J., Liu Y., Banfield J.F., Gu J.D. (2018). Insights into the ecology, evolution, and metabolism of the widespread Woesearchaeotal lineages. Microbiome.

[34] Zhou Z., Pan J., Wang F., Gu J.D., Li M. (2018). Bathyarchaeota: Globally distributed metabolic generalists in anoxic environments. FEMS Microbiol. Rev..

[35] Price M.N., Dehal P.S., Arkin A.P. (2009). FastTree: Computing large minimum evolution trees with profiles instead of a distance matrix. Mol. Biol. Evol..

[36] McMurdie P.J., Holmes S. (2013). phyloseq: An R package for reproducible interactive analysis and graphics of microbiome census data. PLoS ONE.

[37] Oksanen J., Blanchet F.G., Kindt R., Legendre P., Minchin P.R., O’hara R.B., Simpson G.L., Solymos P., Steven H.M.H., Szoecs E. (2013). Package ‘Vegan’: Community Ecology Package. R Found. Stat. Comput..

[38] Pereira M.B., Wallroth M., Jonsson V., Kristiansson E. (2018). Comparison of normalization methods for the analysis of metagenomic gene abundance data. BMC Genom..

[39] Faith D.P. (1992). Conservation evaluation and phylogenetic diversity. Biol. Conserv.

[40] Webb C.O., Ackerly D.D., Kembel S.W. (2008). Phylocom: Software for the analysis of phylogenetic community structure and trait evolution. Bioinformatics.

[41] Mazel F., Davies T.J., Gallien L., Renaud J., Groussin M., Münkemüller T., Thuiller W. (2016). Influence of tree shape and evolutionary time-scale on phylogenetic diversity metrics. Ecography.

[42] Kembel S.W., Cowan P.D., Helmus M.R., Cornwell W.K., Morlon H., Ackerly D.D., Blomberg S.P., Webb C.O. (2010). Picante: R tools for integrating phylogenies and ecology. Bioinformatics.

[43] Kembel S.W., Eisen J.A., Pollard K.S., Green J.L. (2011). The phylogenetic diversity of metagenomes. PLoS ONE.

[44] Tripathi B.M., Kim M., Kim Y., Byun E., Yang J.W., Ahn J., Lee Y.K. (2018). Variations in bacterial and archaeal communities along depth profiles of Alaskan soil cores. Sci. Rep..

[45] Baldrian P., Kolařík M., Štursová M., Kopecký J., Valášková V., Větrovský T., Žifčáková L., Šnajdr J., Rídl J., Vlček C. (2012). Active and total microbial communities in forest soil are largely different and highly stratified during decomposition. ISME J..

[46] Ko D., Yoo G., Yun S.T., Jun S.C., Chung H. (2017). Bacterial and fungal community composition across the soil depth profiles in a fallow field. J. Ecol. Environ..

[47] Feng H., Guo J., Wang W., Song X., Yu S. (2019). Soil depth determines the composition and diversity of bacterial and archaeal communities in a poplar plantation. Forests.

[48] Cao P., Zhang L.M., Shen J.P., Zheng Y.M., Di H.J., He J.Z. (2012). Distribution and diversity of archaeal communities in selected Chinese soils. FEMS Microbiol. Ecol..

[49] Akob D.M., Küsel K. (2011). Where microorganisms meet rocks in the Earth’s Critical Zone. Biogeosciences.

[50] Caruso T., Schaefer I., Monson F., Keith A.M. (2019). Oribatid mites show how climate and latitudinal gradients in organic matter can drive large-scale biodiversity patterns of soil communities. J. Biogeogr..

[51] Tucker C.M., Cadotte M.W., Carvalho S.B., Davies T.J., Ferrier S., Fritz S.A., Grenyer R., Helmus M.R., Jin L.S., Mooers A.O. (2017). A guide to phylogenetic metrics for conservation, community ecology and macroecology. Biol. Rev..

[52] Herrmann M., Wegner C.E., Taubert M., Geesink P., Lehmann K., Yan L., Lehmann R., Totsche K.U., Küsel K. (2019). Predominance of Cand. Patescibacteria in groundwater is caused by their preferential mobilization from soils and flourishing under oligotrophic conditions. Front. Microbiol..

[53] Anacker B.L., Harrison S.P. (2012). Historical and ecological controls on phylogenetic diversity in Californian plant communities. Am. Nat..

[54] Abu-Ashour J., Joy D.M., Lee H., Whiteley H.R., Zelin S. (1994). Transport of microorganisms through soil. Water Air Soil Pollut..

[55] Hamarashid N.H., Othman M.A., Hussain M.A.H. (2010). Effects of soil texture on chemical compositions, microbial populations and carbon mineralization in soil. Egypt J. Exp. Biol..

[56] Griffiths R.I., Whiteley A.S., O’Donnell A.G., Bailey M.J. (2003). Influence of depth and sampling time on bacterial community structure in an upland grassland soil. FEMS Microbiol. Ecol..

[57] Li X., Wang H., Li X., Li X., Zhang H. (2019). Shifts in bacterial community composition increase with depth in three soil types from paddy fields in China. Pedobiologia.

[58] Jones R.T., Robeson M.S., Lauber C.L., Hamady M., Knight R., Fierer N. (2009). A comprehensive survey of soil acidobacterial diversity using pyrosequencing and clone library analyses. ISME J..

[59] Govil T., Rathinam N.K., Salem D.R., Sani R.K. (2019). Taxonomical diversity of extremophiles in the deep biosphere. Microbial Diversity in the Genomic Era.

[60] Bräuer S.L., Cadillo-Quiroz H., Ward R.J., Yavitt J.B., Zinder S.H. (2011). Methanoregula boonei gen. nov., sp. nov., an acidiphilic methanogen isolated from an acidic peat bog. Int. J. Syst. Evol. Microbiol..

[61] Mondav R., Woodcroft B.J., Kim E.H., McCalley C.K., Hodgkins S.B., Crill P.M., Chanton J., Hurst G.B., VerBerkmoes N.C., Saleska S.R. (2014). Discovery of a novel methanogen prevalent in thawing permafrost. Nat. Commun..

[62] Haroon M.F., Hu S., Shi Y., Imelfort M., Keller J., Hugenholtz P., Tyson G.W. (2013). Anaerobic oxidation of methane coupled to nitrate reduction in a novel archaeal lineage. Nature.

[63] Li Y., Adams J., Shi Y., Wang H., He J.S., Chu H. (2017). Distinct Soil Microbial Communities in habitats of differing soil water balance on the Tibetan Plateau. Sci. Rep..

[64] Shen C., Liang W., Shi Y., Lin X., Zhang H., Wu X., Xie G., Chain P., Grognan P., Chu H. (2014). Contrasting elevational diversity patterns between eukaryotic soil microbes and plants. Ecology.

[65] Kang B., Bowatte S., Hou F. (2021). Soil microbial communities and their relationships to soil properties at different depths in an alpine meadow and desert grassland in the Qilian mountain range of China. J. Arid Environ..

[66] Chen H., Yang Z.K., Yip D., Morris R.H., Lebreux S.J., Cregger M.A., Klingeman D.M., Hui D., Hettich R.L., Wilhelm S.W. (2019). One-time nitrogen fertilization shifts switchgrass soil microbiomes within a context of larger spatial and temporal variation. PLoS ONE.

[67] Borgonie G., García-Moyano A., Litthauer D., Bert W., Bester A., van Heerden E., Moller C., Erasmus M., Onstott T.C. (2011). Nematoda from the terrestrial deep subsurface of South Africa. Nature.

[68] Wan X., Gao Q., Zhao J., Feng J., van Nostrand J.D., Yang Y., Zhou J. (2020). Biogeographic patterns of microbial association networks in paddy soil within Eastern China. Soil Biol. Biochem..

[69] Mackenzie B.W., Chang K., Zoing M., Jain R., Hoggard M., Biswas K., Douglas R.G., Taylor M.W. (2019). Longitudinal study of the bacterial and fungal microbiota in the human sinuses reveals seasonal and annual changes in diversity. Sci. Rep..

[70] Kneip C., Lockhart P., Voβ C., Maier U.G. (2007). Nitrogen fixation in eukaryotes–new models for symbiosis. BMC Evol. Biol..

[71] Ibelings B.W., De Bruin A., Kagami M., Rijkeboer M., Brehm M., Donk E.V. (2004). Host parasite interactions between freshwater phytoplankton and chytrid fungi (chytridiomycota) 1. J. Phycol..

[72] Kieft T.L., Murphy E.M., Haldeman D.L., Amy P.S., Bjornstad B.N., McDonald E.V., Ringelberg D.B., White D.C., Stair J., Griffiths R.P. (1998). Microbial transport, survival, and succession in a sequence of buried sediments. Microb. Ecol..

[73] Rinke C., Rubino F., Messer L.F., Youssef N., Parks D.H., Chuvochina M., Brown M., Jeffries T., Tyson G.W., Seymour J.R. (2019). A phylogenomic and ecological analysis of the globally abundant Marine Group II archaea (Ca. Poseidoniales ord. nov.). ISME J..

[74] Cavalier-Smith T., Chao E.E., Thompson C.E., Hourihane S.L. (1996). Oikomonas, a distinctive zooflagellate related to chrysomonads. Arch. Protistenkd..

[75] Griebler C., Mindl B., Slezak D., Geiger-Kaiser M. (2002). Distribution patterns of attached and suspended bacteria in pristine and contaminated shallow aquifers studied with an in situ sediment exposure microcosm. Aquat. Microb. Ecol..

