# Supplementary material for: From Surface to Subsurface: Diversity, Composition, and Abundance of Sessile and Endolithic Bacterial, Archaeal, and Eukaryotic Communities in Sand, Clay and Rock Substrates in the Laurentians (Quebec, Canada)
Source: Microorganisms. 2022 Jan 8;10(1):129. doi: 10.3390/microorganisms10010129 (PMC8781179; doi:10.3390/microorganisms10010129)
Supplement: Supplementary file 1 [file microorganisms-10-00129-s001.zip › microorganisms-1469595-supplementary.pdf]

SUPPLEMENTARY FIGURES

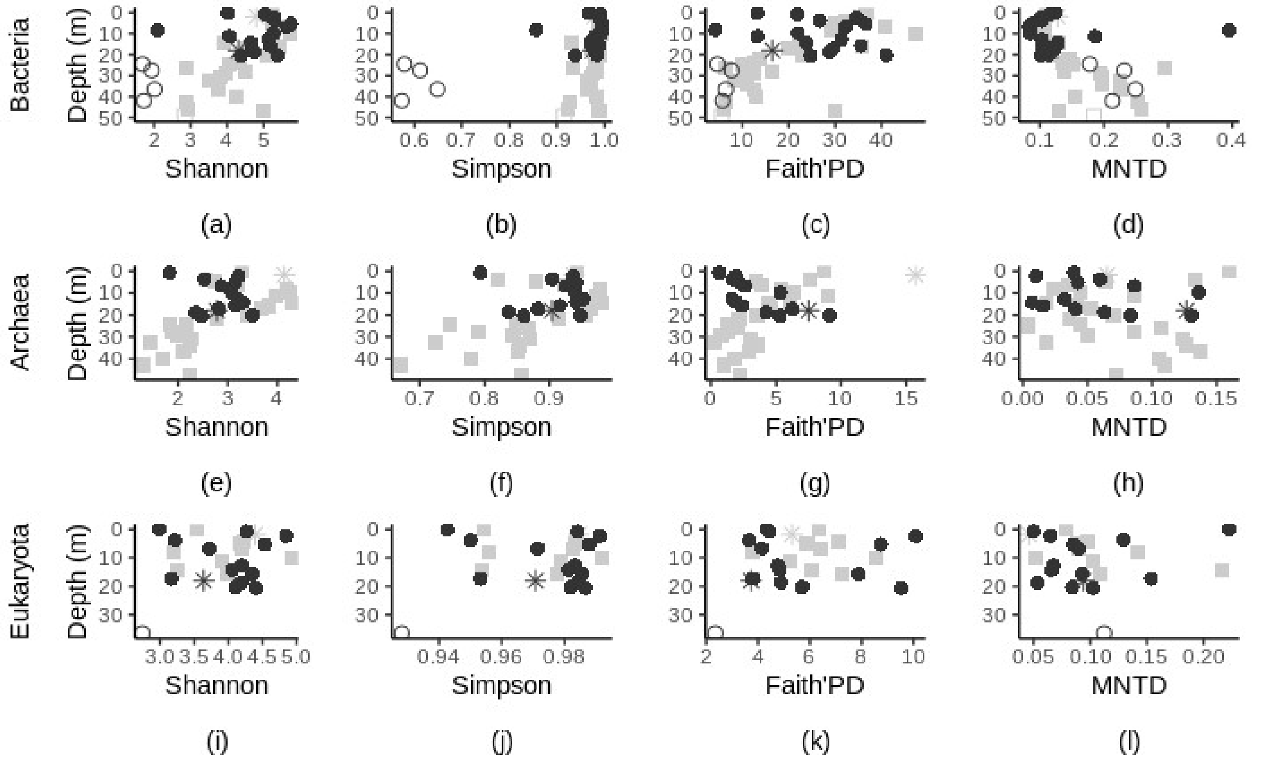

Figure S1. Bacterial, archaeal and eukaryotic α-diversity indexes along depth (m) at site 1 (black, circle) and site 2 (grey, square). With: (**a**,**e**,**i**) Shannon; (**b**,**f**,**j**) Simpson index; (**c**,**g**,**k**) Faith's PD and (**d**,**h**,**l**) MNTD index. Bedrock samples are represented by hollow symbols. Groundwater samples are represented by stars.

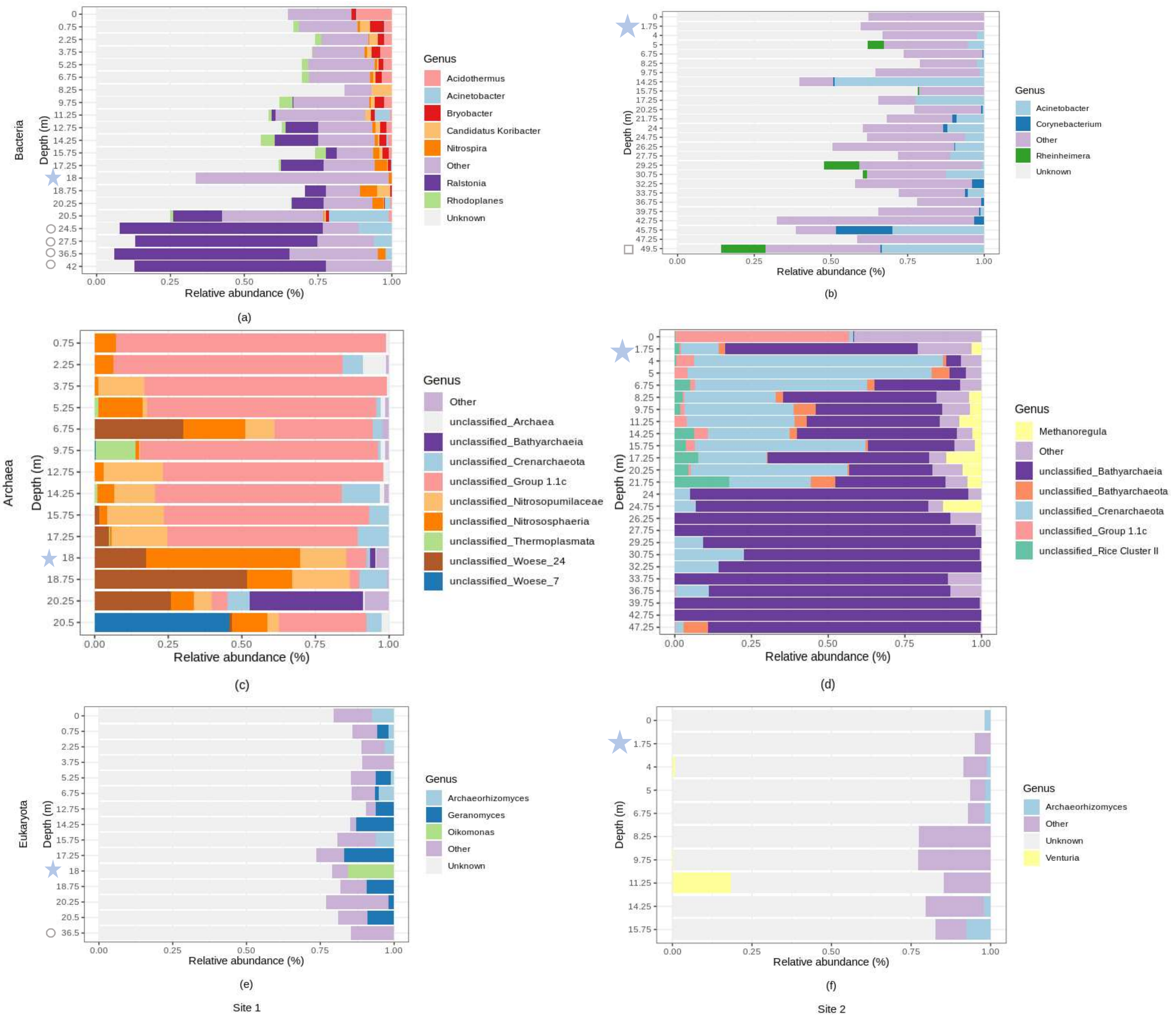

Figure S2. Relative abundance (%) of each microbial genera across different depths (m) at site 1 and at site 2. With: (**a**) bacterial relative abundance at site 1, (**b**) bacterial relative abundance at site 2, (**c**) archaeal relative abundance at site 1, (**d**) archaeal relative abundance at site 2, (**e**) eukaryotic relative abundance at site 1, (**f**) eukaryotic relative abundance at site 2. Genera with an average relative abundance of less than 1 % were categorized as "Other". Bedrock samples are represented by hollow circles and hollow squares. Groundwater samples are represented by blue stars.

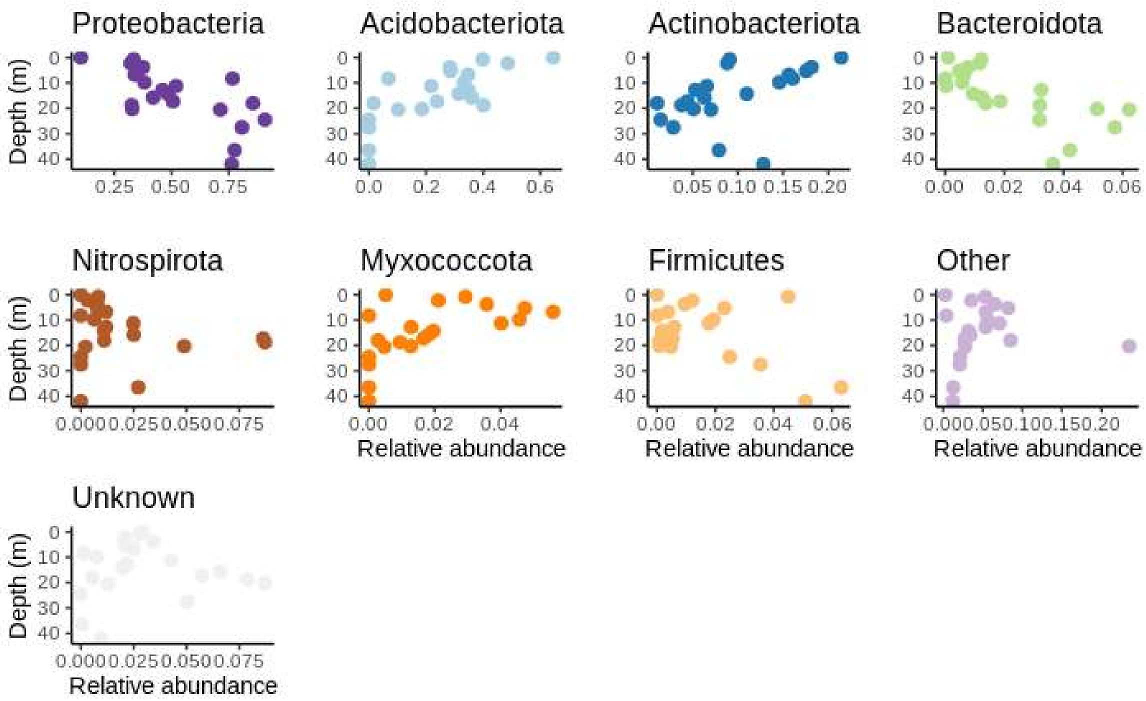

Figure S3. Change in the relative abundance of most abundant bacterial phyla with depth at site 1.

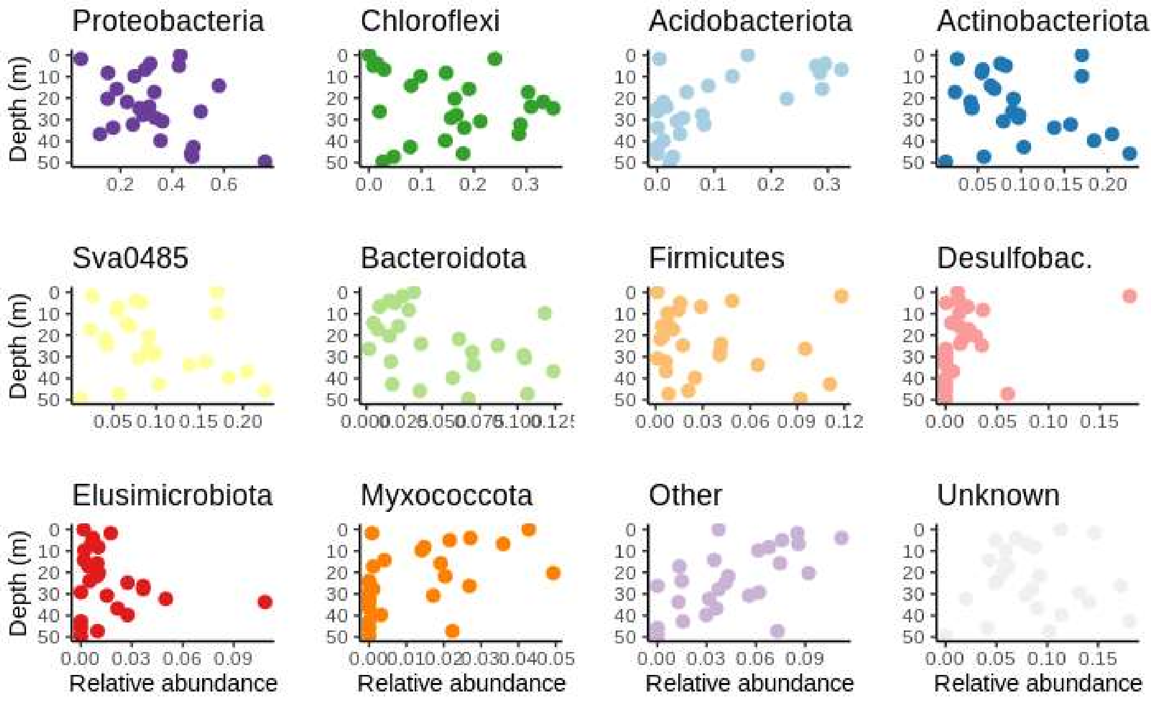

Figure S4. Change in the relative abundance of most abundant bacterial phyla with depth at site 2.

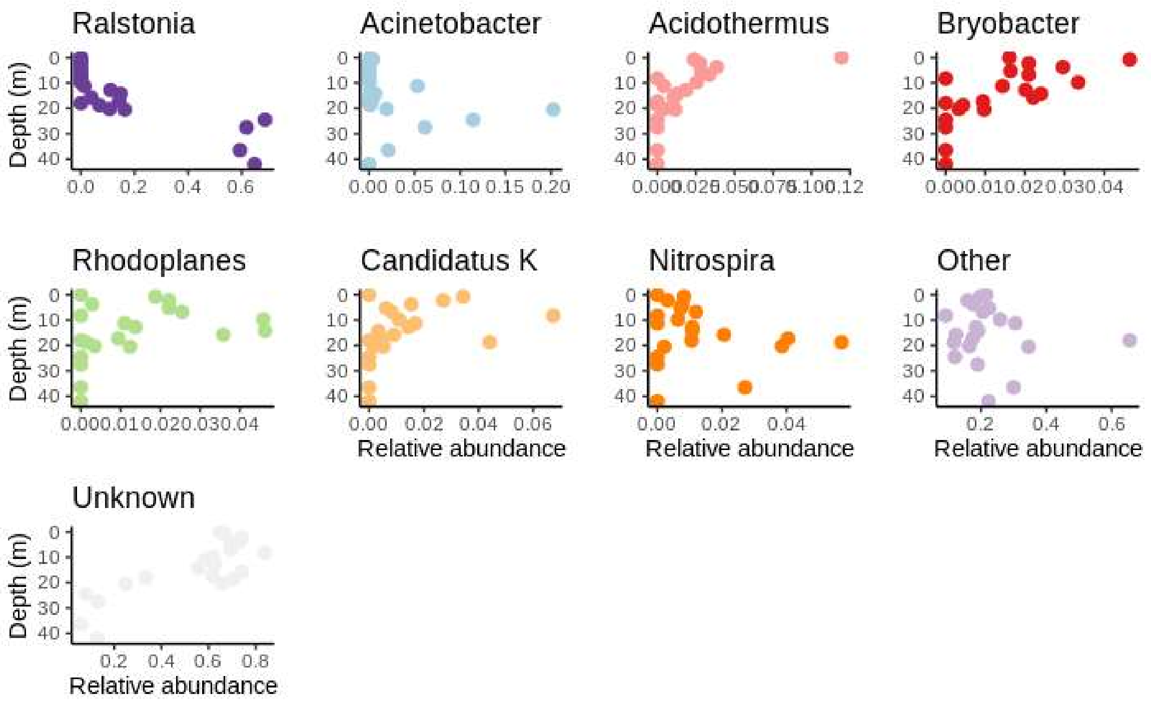

Figure S5. Change in the relative abundance of most abundant bacterial genera with depth at site 1.

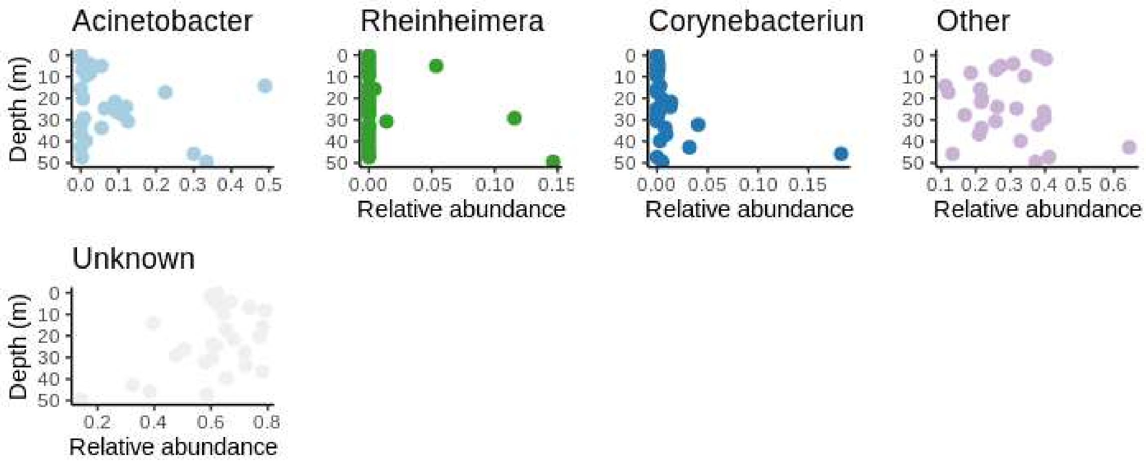

Figure S6. Change in the relative abundance of most abundant bacterial genera with depth at site 2.

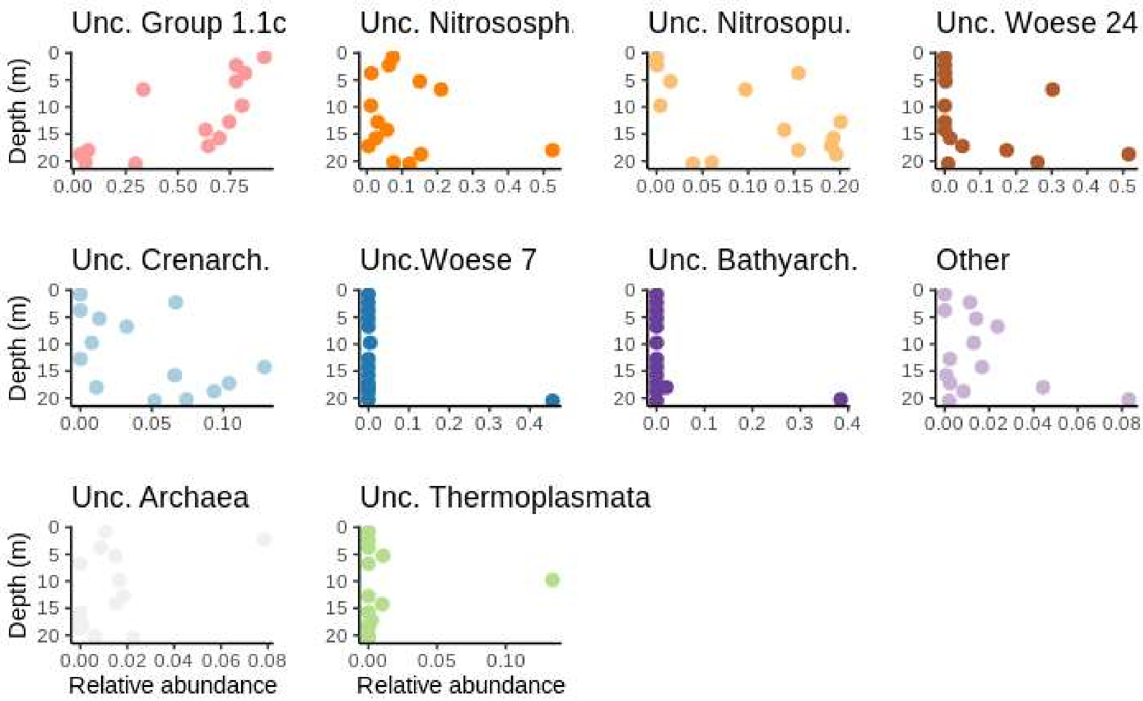

Figure S7. Change in the relative abundance of most abundant archaeal genera with depth at site 1. Unc : uncultured.

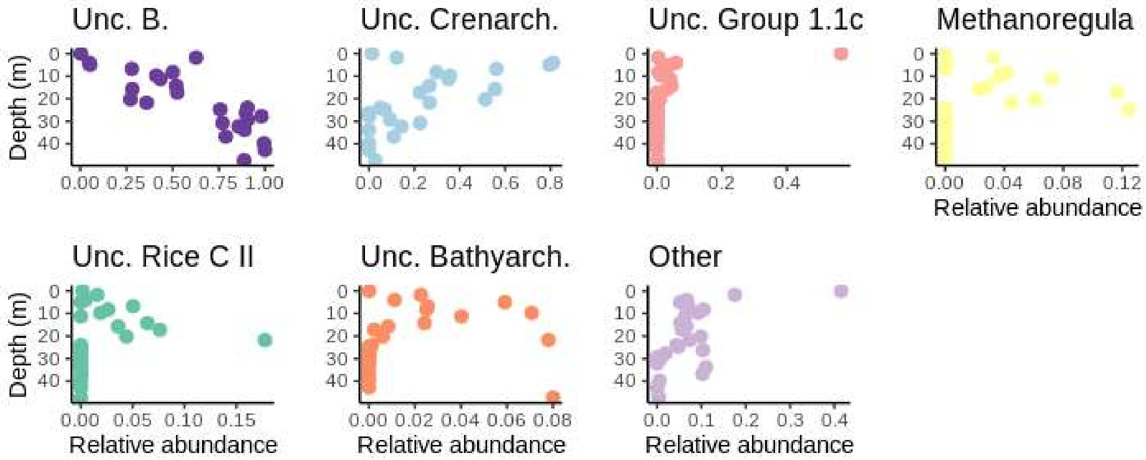

Figure S8. Change in the relative abundance of most abundant archaeal genera with depth at site 2. Unc. B : Uncultured Bathyarchaeia.

SUPPLEMENTARY TABLES

Table S1: Abiotic characteristics measured along depth gradient at site 1.

Sample	Depth (m)	Texture	pH	TN (%)	TC (%)	TOC (%)	TIC (%)
**RR-01**	0	mS	5.16 ± 0.20	1.52	48.24	30.73	17.51
**RR-02**	0.75	mS	6.53 ± 0.20	0.00	0.04	0.03	0.01
**RR-03**	2.25	mS	6.43 ± 0.09	0.00	0.05	0.04	0.01
**RR-04**	3.75	mcSG	6.45 ± 0.13	0.00	0.03	0.03	0.00
**RR-05**	5.25	mcSG	6.88 ± 0.09	0.00	0.03	0.02	0.01
**RR-06**	6.75	mcSG	6.84 ± 0.10	0.00	0.03	0.02	0.01
**RR-07**	8.25	mcSG	6.78 ± 0.05	0.00	0.02	0.00	0.02
**RR-08**	9.75	mcSG	6.66 ± 0.06	0.00	0.02	0.02	0.00
**RR-09**	11.25	mcSG	6.62 ± 0.20	0.00	0.06	0.06	0.00
**RR-10**	12.75	cSG	6.78 ± 0.22	0.00	0.03	0.02	0.01
**RR-11**	14.25	cSG	6.93 ± 0.08	0.00	0.01	0.00	0.01
**RR-12**	15.75	cSG	7.07 ± 0.07	0.00	0.02	0.00	0.02
**RR-13**	17.25	cSG	6.43 ± 0.15	0.00	0.01	0.00	0.01
**RR-14**	18.75	cSG	6.98 ± 0.09	0.00	0.02	0.00	0.02
**RR-15**	20.25	cSG	8.11 ± 0.09	0.00	0.02	0.00	0.02
**RR-16**	20.5	cSG	8.58 ± 0.31	0.00	0.01	0.00	0.01
**RR-17**	24.5	R	9.14 ± 0.05	0.00	0.04	0.00	0.04
**RR-18**	27.5	R	9.44 ± 0.07	0.00	0.02	0.00	0.02
**RR-19**	36.5	R	9.41 ± 0.04	0.00	0.01	0.00	0.01
**RR-20**	42	R	9.53 ± 0.06	0.00	0.05	0.00	0.05
**RR-eau-puit**		GW	7.23	-	-	-	-

mS, medium sand; mcSG, medium to coarse sand and gravel; cSG, coarse sand and gravel; R, Bedrock; GW, groundwater. ± report the standard deviation. 0.00 is for values that were below the limit of detection (LOD).

Table S2: Abiotic characteristics measured along depth gradient at site 2.

Sample	Depth (m)	Texture	pH	TN (%)	TC (%)	TOC (%)	TIC (%)
**NDDL-01**	0	fmS	6.23 ± 0.03	0.03	0.48	0.43	0.05
**NDDL-02**	4	fmS	6.93 ± 0.01	0.01	0.17	0.16	0.01
**NDDL-03**	5	fmS	6.76 ± 0.02	0.01	0.18	0.18	0.00
**NDDL-04**	6.75	fmS	6.95 ± 0.02	0.00	0.12	0.12	0.00
**NDDL-05**	8.25	fvfS	7.56 ± 0.02	0.00	0.02	0.02	0.00
**NDDL-06**	9.75	fvfS	8.36 ± 0.03	0.00	0.03	0.03	0.00
**NDDL-07**	11.25	fvfS	8.96 ± 0.02	0.00	0.47	0.03	0.44
**NDDL-08**	14.25	fvfS	9.07 ± 0.03	0.00	0.42	0.02	0.40
**NDDL-09**	15.75	fvfS	8.66 ± 0.03	0.00	0.59	0.04	0.55
**NDDL-10**	17.25	fvfS	9.30 ± 0.01	0.00	0.43	0.01	0.42
**NDDL-11**	20.25	fvfS	9.35 ± 0.02	0.00	0.42	0.01	0.41
**NDDL-12**	21.75	fvfS	8.97 ± 0.03	0.00	0.52	0.02	0.50
**NDDL-13**	24	fvfS	9.10 ± 0.04	0.00	0.47	0.01	0.46
**NDDL-14**	24.75	fvfS	8.96 ± 0.01	0.00	0.51	0.02	0.49
**NDDL-15**	26.25	fvfS	8.96 ± 0.03	0.00	0.52	0.01	0.51
**NDDL-16**	27.75	fvfS	9.05 ± 0.01	0.00	0.52	0.01	0.51
**NDDL-17**	29.25	fvfS	9.00 ± 0.01	0.00	0.53	0.00	0.53
**NDDL-18**	30.75	SI	8.94 ± 0.02	0.00	0.57	0.02	0.55
**NDDL-19**	32.25	SI	8.75 ± 0.02	0.00	0.52	0.02	0.50
**NDDL-20**	33.75	SI	8.54 ± 0.04	0.00	0.58	0.03	0.55
**NDDL-21**	36.75	SIC	8.46 ± 0.01	0.00	0.67	0.09	0.58
**NDDL-22**	39.75	SIC	8.37 ± 0.03	0.00	1.04	0.09	0.95
**NDDL-23**	42.75	fSSIC	8.66 ± 0.03	0.00	1.25	0.03	1.22
**NDDL-24**	45.75	SG	8.65 ± 0.01	0.00	1.66	0.03	1.63
**NDDL-25**	47.25	SG	8.89 ± 0.05	0.00	2.93	2.27	0.65
**NDDL-26**	49.5	R	8.95 ± 0.01	0.00	1.80	0.46	1.34
**NDDL-eau-puit**		GW	7.02	-	-	-	-

fmS, fine to medium sand; fvfS, fine to very fine sand; SI, silt; SIC, Silt and clay; fSSIC, fine sand with silt and clay; SG, sand and gravel; R, Bedrock; GW, groundwater ± report the standard deviation. 0.00 is for values that were below the limit of detection (LOD).

Table S3: The correlations (r) determined by Spearman Correlation between abiotic characteristics and depth at site 1 and site 2.

	pH	TN	TC	TOC	TIC
**Depth (Site 1)**	**0.83*****	-	**-0.37**	**-0.79*****	**0.58***
**Depth (Site 2)**	0.16	-	**0.91*****	0.07	**0.92*****

Values in bold are significant at p-value <0.05. TN, total nitrogen; TC, total carbon; TOC, total organic carbon; TIC, total inorganic carbon. Signification codes: 0 '***'; 0.01 '*'. Only rocks and geological material from the subsurface were used for correlation.

Table S4: XRD results showing the mineralogical composition (in %) of the samples collected at site 1.

Sample	Quartz	K-feldspar	Plagioclase	Calcite	Dolomite	Halite	Apatite	Pyrite	Olivine	Amphibole	Pyroxene	Organic matter	Kaolinite	Chlorite	Biotite	Illite
**RR-01**	1.5	4.6	2.6	0.0	0.3	0.1	0.5	1.5	1.2	0.8	1.1	84.9	0.6	0.0	0.0	0.0
**RR-02**	45.2	22.3	32.3	0.0	0.0	0.0	0.0	0.2	0.0	0.0	0.0	0.0	0.0	0.0	0.0	0.0
**RR-03**	36.0	21.3	42.6	0.0	0.0	0.0	0.0	0.1	0.0	0.0	0.0	0.0	0.0	0.0	0.0	0.0
**RR-04**	46.8	19.4	31.6	0.0	0.0	0.1	0.0	0.4	0.6	0.0	0.0	0.0	0.0	0.0	0.0	1.0
**RR-05**	35.7	23.1	40.5	0.0	0.0	0.0	0.0	0.8	0.0	0.0	0.0	0.0	0.0	0.0	0.0	0.0
**RR-06**	49.8	19.7	27.7	0.0	0.0	0.0	0.0	1.0	1.0	0.0	0.0	0.0	0.0	0.0	0.0	0.7
**RR-07**	47.2	22.1	26.8	0.0	0.0	0.3	0.9	0.8	1.0	0.0	0.0	0.0	0.0	0.1	0.0	0.8
**RR-08**	49.9	21.1	28.7	0.0	0.0	0.0	0.0	0.3	0.0	0.0	0.0	0.0	0.0	0.0	0.0	0.0
**RR-09**	44.0	22.7	33.0	0.0	0.0	0.0	0.0	0.3	0.0	0.0	0.0	0.0	0.0	0.0	0.0	0.0
**RR-10**	44.6	10.0	39.9	0.0	0.0	0.0	0.0	0.2	0.0	0.6	0.1	0.0	0.0	0.0	1.6	3.0
**RR-11**	31.4	24.2	41.9	0.0	0.0	0.0	0.0	0.3	0.0	0.0	0.0	0.0	0.0	2.3	0.0	0.0
**RR-12**	32.5	19.0	45.2	0.0	0.0	0.0	0.0	0.6	0.0	0.0	1.2	0.0	0.0	0.0	0.0	1.6
**RR-13**	34.4	17.6	47.4	0.0	0.0	0.0	0.0	0.2	0.0	0.0	0.0	0.0	0.0	0.2	0.0	0.3
**RR-14**	48.6	14.7	34.6	0.0	0.0	0.0	0.0	0.1	0.0	0.0	1.9	0.0	0.0	0.0	0.0	0.0
**RR-15**	34.0	23.1	37.6	0.0	0.0	0.3	0.0	0.5	1.1	2.6	0.0	0.0	0.0	0.0	0.0	0.8
**RR-16**	30.4	12.0	48.5	0.0	0.0	0.2	0.0	0.4	1.4	0.0	0.0	0.0	0.0	0.0	7.1	0.0
**RR-17**	31.3	8.1	57.9	0.0	0.0	0.0	0.0	0.3	2.4	0.0	0.0	0.0	0.0	0.0	0.0	0.0
**RR-18**	24.7	27.9	46.3	0.0	0.0	0.0	0.0	0.1	0.0	0.0	0.4	0.0	0.0	0.0	0.5	0.0
**RR-19**	28.2	23.3	45.9	0.0	0.0	0.0	0.0	0.0	0.0	0.0	0.8	0.0	0.0	0.0	0.4	1.4
**RR-20**	30.4	25.3	44.3	0.0	0.0	0.0	0.0	0.0	0.0	0.0	0.0	0.0	0.0	0.0	0.0	0.0

Table S5: XRD results showing the mineralogical composition (in %) of the samples collected at site 2.

Sample	Quartz	K-feldspar	Plagioclase	Calcite	Dolomite	Halite	Apatite	Pyrite	Olivine	Amphibole	Pyroxene	Organic matter	Kaolinite	Chlorite	Biotite	Illite
**NDDL-01**	50.8	23.9	20.3	0.0	0.0	0.1	0.6	0.8	0.0	0.0	3.5	0.0	0.0	0.0	0.0	0.0
**NDDL-02**	37.9	19.0	37.2	0.0	0.0	1.0	0.0	0.0	4.9	0.0	0.0	0.0	0.0	0.0	0.0	0.0
**NDDL-03**	32.8	17.8	33.8	0.0	0.0	0.2	0.0	1.4	2.1	1.4	3.1	0.0	1.9	0.0	0.0	5.6
**NDDL-04**	38.4	24.5	36.3	0.0	0.0	0.0	0.0	0.9	0.0	0.0	0.0	0.0	0.0	0.0	0.0	0.0
**NDDL-05**	30.0	27.9	23.9	0.0	0.0	0.0	0.1	1.6	2.6	0.0	9.7	0.0	0.0	0.1	0.0	4.1
**NDDL-06**	45.1	21.0	31.4	0.0	0.0	0.0	0.0	0.4	0.0	0.0	1.1	0.0	0.0	0.0	0.0	1.0
**NDDL-07**	34.0	21.9	38.2	0.0	1.7	0.0	0.0	4.1	0.0	0.0	0.0	0.0	0.0	0.0	0.0	0.0
**NDDL-08**	45.7	16.8	32.4	0.9	0.0	0.0	0.0	0.0	0.6	0.0	1.2	0.0	0.0	0.0	0.0	2.4
**NDDL-09**	32.1	18.1	39.1	2.5	0.0	0.0	0.0	0.0	3.3	1.5	0.0	0.8	0.4	0.3	0.6	1.3
**NDDL-10**	52.0	14.4	26.6	0.0	2.1	0.0	0.0	2.5	1.2	0.0	0.0	0.0	0.0	0.0	0.0	1.2
**NDDL-11**	27.0	30.0	40.7	0.0	0.9	0.0	0.0	1.4	0.0	0.0	0.0	0.0	0.0	0.0	0.0	0.0
**NDDL-12**	40.4	18.5	32.8	3.5	0.0	0.0	0.0	0.9	0.0	0.0	3.0	0.0	0.0	1.0	0.0	0.0
**NDDL-13**	29.9	30.8	34.8	3.2	0.0	0.0	0.0	1.3	0.1	0.0	0.0	0.0	0.0	0.0	0.0	0.0
**NDDL-14**	45.3	8.0	43.8	0.0	0.0	1.5	0.0	0.7	0.7	0.0	0.0	0.0	0.0	0.0	0.0	0.0
**NDDL-15**	43.2	13.6	39.7	0.0	1.1	0.0	0.0	1.5	0.0	0.0	0.0	0.0	0.9	0.0	0.0	0.0
**NDDL-16**	26.8	16.5	55.0	0.8	0.0	0.0	0.0	0.9	0.0	0.0	0.0	0.0	0.0	0.0	0.0	0.0
**NDDL-17**	29.9	16.3	47.3	0.0	0.0	0.0	0.8	2.0	2.7	0.0	0.0	0.0	0.0	1.0	0.0	0.0
**NDDL-18**	26.0	24.4	41.8	2.7	0.0	0.0	0.0	1.0	0.0	0.0	1.8	0.0	0.0	0.0	0.0	2.2
**NDDL-19**	27.4	21.3	43.2	0.0	0.7	0.0	0.2	1.0	0.0	0.0	6.2	0.0	0.0	0.0	0.0	0.0
**NDDL-20**	22.4	22.1	46.8	1.8	0.3	0.0	0.2	0.0	1.2	0.5	4.1	0.0	0.0	0.0	0.6	0.0
**NDDL-21**	23.7	19.3	44.2	3.1	0.8	0.0	0.0	1.9	0.9	2.0	4.1	0.0	0.0	0.0	0.0	0.0
**NDDL-22**	19.2	19.5	34.6	4.2	0.8	0.0	0.4	1.9	1.5	5.6	7.6	0.0	0.4	0.0	0.5	3.7
**NDDL-23**	50.7	7.8	34.0	0.0	2.5	0.0	0.0	3.0	1.6	0.0	0.0	0.0	0.0	0.0	0.4	0.0
**NDDL-24**	35.1	25.8	26.1	10.3	2.4	0.3	0.0	0.0	0.0	0.0	0.0	0.0	0.0	0.0	0.0	0.0
**NDDL-25**	34.0	6.0	19.4	29.4	5.2	0.0	0.1	1.1	0.0	0.0	3.2	0.0	0.0	0.0	0.0	1.6
**NDDL-26**	17.2	31.4	28.4	19.9	1.1	0.0	0.0	0.0	0.0	0.0	0.0	0.0	0.0	0.0	0.0	2.1

Table S6: Spearman correlation between bacterial α-diversity metrics and characteristics of geological material at site 1 and site 2.

	Shannon	Simpson	PD	MNTD
**Site 1**				
**Depth**	**-0.54**	**-0.67***	-0.28	0.42
**pH**	-0.40	**-0.51**	-0.23	0.30
**TN**	-	-	-	-
**TC**	-0.00	0.13	-0.12	-0.02
**TOC**	0.38	**0.54**	0.14	-0.36
**TIC**	**-0.47**	**-0.57***	-0.24	0.43
**Site 2**				
**Depth**	**-0.77*****	**-0.62***	**-0.75*****	**0.80*****
**pH**	-0.33	-0.26	-0.38	0.26
**TN**	-	-	-	-
**TC**	**-0.65****	**-0.50**	**-0.59***	**0.73*****
**TOC**	0.18	0.15	0.30	-0.21
**TIC**	**-0.71*****	**-0.58***	**-0.67****	**0.79*****

Values in bold are significant at p-value < 0.05. TN: total nitrogen; TC: total carbon; TOC: total organic carbon; TIC: total inorganic carbon. Signification codes: 0 '***'; 0.001 '**'; 0.01 '*'; 0.05 '.'.

Table S7: Spearman Correlation between archaeal α-diversity metrics and characteristics of geological material at site 1 and site 2.

	Shannon	Simpson	PD	MNTD
**Site 1**				
**Depth**	0.06	-0.01	**0.73***	0.30
**pH**	0.13	0.10	0.38	0.27
**TN**	-	-	-	-
**TC**	-0.01	0.08	**-0.67**	-0.20
**TOC**	-0.08	0.03	**-0.67**	-0.12
**TIC**	0.16	-0.03	0.28	-0.19
**Site 2**				
**Depth**	**-0.73*****	**-0.62***	**-0.73*****	0.20
**pH**	-0.07	0.01	-0.37	-0.27
**TN**	-	-	-	-
**TC**	**-0.62***	**-0.55***	**-0.51**	0.38
**TOC**	0.12	0.03	**0.42**	0.31
**TIC**	**-0.65****	**-0.57***	**-0.55***	0.36

Values in bold are significant at p-value < 0.05. TN: total nitrogen; TC: total carbon; TOC: total organic carbon; TIC: total inorganic carbon. Signification codes: 0 '***'; 0.001 '**'; 0.01 '*'; 0.05 '.'.

Table S8: Spearman Correlation between eukaryotic α-diversity metrics and characteristics of geological material at site 1 and site 2.

	Shannon	Simpson	PD	MNTD
**Site 1**				
**Depth**	-0.13	-0.09	0.03	0.11
**pH**	0.11	0.12	0.22	-0.12
**TN**	-	-	-	-
**TC**	0.22	0.17	0.07	-0.18
**TOC**	0.09	0.03	-0.08	-0.01
**TIC**	-0.03	0.03	0.27	-0.02
**Site 2**				
**Depth**	-0.2	-0.23	0.07	0.58
**pH**	-0.23	-0.25	-0.03	0.63
**TN**	-	-	-	-
**TC**	-0.23	-0.43	0.08	0.12
**TOC**	0.39	0.27	0.25	-0.66
**TIC**	-0.32	-0.48	0.06	0.45

Values in bold are significant at p-value < 0.05. TN: total nitrogen; TC: total carbon; TOC: total organic carbon; TIC: total inorganic carbon.

Table S9: Mantel and ANOSIM test between bacteria ß-diversity metrics and characteristics of geological material.

	Depth	pH	TN	TC	TOC	TIC	Mineralogy	Texture
**Bacteria**								
**Site 1**	**0.35***	**0.66****	**-**	0.07	**-**	**-**	**0.26***	**0.64****
**Site 2**	**0.53****	**0.37****	**-**	**0.28****	0.04	**-**	0.04	**0.47****
**Archaea**								
**Site 1**	**0.25**	0.12	**-**	0.14	**-**	**-**	0.07	0.20
**Site 2**	**0.59****	**0.22***	**-**	**0.23****	0.05	**-**	0.08	0.15
**Eukaryote**								
**Site 1**	**0.25***	**0.23***	**-**	**0.31***	**-**	-0.03	0.04	0.30
**Site 2**	**0.27**	0.19	**-**	0.13	**0.37**	**-**	0.15	-0.2

Values in bold are significant at p-value < 0.05. TN: total nitrogen; TC: total carbon; TOC: total organic carbon; TIC: total inorganic carbon. Signification codes: 0.001 '**'; 0.01 '*'; 0.05 '.'.

Table S10: Spearman Correlation between absolute abundance and characteristics of geological material at site 1 and site 2.

**Abundance (copies g^{-1})**	
**Site 1**	
**Depth**	0.12
**pH**	0.01
**TN**	-
**TC**	0.14
**TOC**	0.11
**TIC**	0.57
**Site 2**	
**Depth**	**-0.68***
**pH**	-0.18
**TN**	-
**TC**	**-0.47**
**TOC**	0.08
**TIC**	**-0.53**

Values in bold are significant at p-value < 0.05. TN: total nitrogen; TC: total carbon; TOC: total organic carbon; TIC: total inorganic carbon. Signification codes: 0.01 '*'; 0.05 '.'.

Table S11: Spearman correlation between relative abundances of most abundant bacterial, archaeal, and eukaryotic phyla and genera with characteristics of geological material at site 1 and site 2.

	Depth (site 1)	Depth (site 2)
**Bacterial phyla**		
Proteobacteria	**0.68***	0.23
Acidobacteriota	**-0.72****	**-0.73*****
Actinobacteriota	**-0.65***	0.22
Bacteroidota	**0.77****	0.44
Nitrospirota	0.11	-
Myxococcota	**-0.66***	**-0.62****
Firmicutes	0.28	0.30
Other	-0.34	**-0.56***
Unknow	-0.11	0.11
Chloroflexi	-	0.25
Sva0485	-	-0.08
Desulfobacterota	-	-0.47
Elusimicrobiota	-	0.13
**Bacterial genera**		
Ralstonia	**0.94*****	-
Acinetobacter	0.49	0.08
Acidothermus	**-0.80*****	-
Bryobacter	**-0.70****	-
Rhodoplanes	-0.39	-
Candidatus Kirobacter	-0.55	-
Nitrospira	0.09	-
Rheinheimera	-	0.09
Corynebacterium	-	0.46
Other	0.06	0.23
Unknow	**-0.67***	-0.45
**Archaeal phyla**		
Crenarchaeota	**-0.62**	**0.58***
Woesearchaeota	**0.70***	-0.11
Thermoplasmatota	-0.19	-
Other	**0.61***	-0.19
Unclassified Archaea	-0.20	-
Halobacterota	-	**-0.61***
Bathyarchaeota	-	**-0.41**
**Archaeal genera**		
Unclassified Group 1.1c	**-0.84****	**-0.75*****
Unclassified Nitrososphaeria	0.08	-
Unclassified Nitrosopumilaceae	0.48	-
Unclassified Woese 24	**0.57**	-
Unclassified Crenarchaeota	**0.58**	**-0.70****
Unclassified Woese 7	0.49	-
Unclassified Bathyarchaeia	0.52	**0.84*****

Other	0.18	**-0.5**
Unclassified Archaea	-0.20	-
Unclassified Thermoplasmata	0.09	-
Methanoregula	-	**-0.41**
Unclassified Rice Cluster II	-	**-0.54***
Unclassified Bathyarchaeota	-	**-0.49**
**Eukaryotic phyla**		
Basidiomycota	**-0.82****	0.07
Ascomycota	0.10	0.5
Phragmoplastophyta	-0.41	-0.33
Chytridiomycota	0.48	
Cercozoa	0.31	0.48
Vertebrata	**0.87*****	-
Dinoflagellata	**0.86*****	-
Arthropoda	0.14	0.55
Fungi	0.20	0.12
Nematozoa	**-0.76***	0.08
Annelida	-0.44	-
Ciliophora	0.48	-
Mucoromycota	**0.63**	0.27
Ochrophyta	**0.55**	-
Metazoa	-	0.37
Cryptomycota	-	-0.40
Mollusca	-	0.55
Other	**0.77***	0.23
Unknowm	**0.76***	-0.15
**Eukaryotic genera**		
Geranomyces	0.34	-
Archaeorhizomyces	**-0.64**	-
Oikomonas	NA	-
Venturia	-	0.22
Archaeorhizomyces	-	0.17
Unknown	-0.45	-0.68
Other	0.35	0.65

Values in bold are significant at p-value < 0.05. Signification codes: 0 '***'; 0.001 '**'; 0.01 '*'; 0.05 '.'.
